# Efficacy of omega‐3 fatty acids as a functional food: a multifaceted approach to health reinforcement

**DOI:** 10.1002/jsfa.70346

**Published:** 2025-12-22

**Authors:** Md Faruque Ahmad, Abdulrahman A Alsayegh, Anjum Khanam, Awais Ahmed, António Raposo, Farkad Bantun, Md Zeyaullah, Ahmad O Babalghith, Abdullah F Aldairi, Boshra Mozaffar, Mohammed F Bajahzer, Mohamed H Abdelrahman, Mohammad Intakhab Alam

**Affiliations:** ^1^ Department of Clinical Nutrition College of Nursing and Health Sciences, Jazan University Jazan Saudi Arabia; ^2^ Department of Management Shri JJT University Rajasthan India; ^3^ CBIOS (Research Center for Biosciences and Health Technologies), ECTS (School of Health Sciences and Technologies), Lusófona University Lisbon Portugal; ^4^ Department of Microbiology and Parasitology Faculty of Medicine, Umm Al‐Qura University Makkah Saudi Arabia; ^5^ Department of Basic Medical Science College of Applied Medical Sciences, Khamis Mushayt Campus, King Khalid University (KKU) Abha Saudi Arabia; ^6^ Department of Medical Genetics Faculty of Medicine, Umm Al‐Qura University Makkah Saudi Arabia; ^7^ Department of Clinical Laboratory Sciences Faculty of Applied Medical Sciences, Umm Al‐Qura University Makkah Saudi Arabia; ^8^ Department of Medical Laboratory Technology, Faculty of Nursing and Health Sciences, Jazan University Jazan Saudi Arabia; ^9^ Department of Pharmaceutics College of Pharmacy, Jazan University Jazan Saudi Arabia

**Keywords:** omega‐3s, bioavailability, cancer, cardiovascular disorders, pregnancy, functional foods

## Abstract

Omega‐3 fatty acids (omega‐3s) are polyunsaturated fatty acids linked with numerous health benefits. Omega‐3s exhibit multifaceted activities through various mechanisms. Eicosapentaenoic acid (EPA) alleviates oxidative stress by lowering reactive oxygen species and improving oxidative stress in brain tissues and acts against neurodegenerative disorders. It has the potential to regulate cell membranes, decrease inflammation, improve endothelial activities, reduce triglycerides (TGs), weaken oxidative stress, and encourage cardiovascular, metabolic and immunological health. Docosahexaenoic acid (DHA) modulates mitochondrial activity, enhances mitochondrial biogenesis and facilitates fatty acid oxidation, which lowers lipid accumulation and helps energy metabolism and is effective in obesity. Omega‐3s such as EPA, DHA and alpha‐linolenic acid show anticancer activity, promote apoptosis, decrease the proliferation of cancer cell and enhance chemotherapy efficacy. Moreover, DHA has potential effects during pregnancy and fetal development. Omega‐3s are often inadequate in our diet and play a significant therapeutic function in several disorders. They have been documented by global health specialists as essential constituents of the human diet. Their relevance depends on their ability to act as a nutritional intervention with significant uses in health promotion. Consequently, dietary nutrition is associated with disease prevention and enhanced clinical results. The growing demand for health‐related products and innovations is expected to have an impact on developing dietary patterns. As a result, omega‐3s are increasingly incorporated into functional foods and supplements. This analysis highlights omega‐3s ability to improve health outcomes as well as prevent chronic diseases. © 2025 The Author(s). *Journal of the Science of Food and Agriculture* published by John Wiley & Sons Ltd on behalf of Society of Chemical Industry.

## INTRODUCTION

A class of polyunsaturated fatty acids known as omega‐3 fatty acids (omega‐3s) has attracted a lot of interest recently because of possible health advantages.^1^ These vital nutrients, which include docosahexaenoic acid (DHA), eicosapentaenoic acid (EPA) and alpha‐linolenic acid (ALA), have been connected to a number of health outcomes and are needed for sustaining a variety of body functions.^2^, ^3^ ALA is a plant‐based omega‐3 fatty acid that is important for human health. Unlike EPA and DHA, ALA cannot be produced by the body and must be taken through food. It acts as a precursor for longer‐chain omega‐3s but has limited conversion efficiency.^4^ For vegetarians, ALA serves as the sole precursor for DHA. DHA can be synthesized from ALA in the body.^5^ This process is significantly rate‐limited in humans, with very limited conversion rates of EPA and DHA, resulting in relatively less formation of EPA and DHA in the body. EPA and DHA are prevalent in marine sources such as fish and fish oils, whereas ALA is mostly found in plant oils.^1^, ^2^, ^6^, ^7^, ^8^, ^9^ EPA is synthesized in lesser amounts from ALA to other omega‐3s. EPA can be transformed into other bioactive substances, such as eicosanoids, which are signaling molecules. EPA is identified for its anti‐inflammatory action, which contributes to decreasing the risk of chronic diseases.^10^, ^11^ DHA is derived from EPA through elongation to docosapentaenoic acid, followed by further elongation, desaturation and retroconversion in peroxisomes to DHA. The metabolic process is inefficient in humans.^12^ DHA is predominantly integrated into phospholipids, including phosphatidylethanolamine and phosphatidylcholine, within cellular membranes.^13^ The elevated level of unsaturation enhances membrane fluidity, which is essential for numerous physiological functions, including protein–lipid interactions and cell signaling.^14^, ^15^ A previous study indicated that DHA enhances membrane fluidity more significantly than EPA.^8^ The interaction of DHA with other membrane lipids, especially cholesterol, influences the configuration and functionality of cell membranes.^15^

Omega‐3s have a wide range of health advantages, including protection against neurological disorders, inflammation and cardiovascular disease. Although recent studies have produced conflicting findings about their effectiveness in avoiding cardiovascular events, omega‐3s have been demonstrated to improve lipid profiles and lower the risk of coronary heart disease for cardiovascular health.^16^, ^17^, ^18^, ^19^ By blocking inflammatory pathways and encouraging the synthesis of anti‐inflammatory mediators, omega‐3s can regulate inflammation, a major contributing factor to many chronic diseases.^20^, ^21^ Additionally, omega‐3s contribute to neuroprotection, which may slow the development of neurodegenerative illnesses such as Parkinson's and Alzheimer's diseases. They are a viable nutritional approach for treating these disorders because of their role in cell membrane fluidity and anti‐inflammatory processes10. Omega‐3s are also essential for fetal development throughout pregnancy, which results in healthy gestations and better perinatal findings.^18^, ^22^, ^23^ To ensure proper intake, health organizations advise including fatty fish and plant‐based sources in the diet. As more research reveals the intricate roles of omega‐3s, this review of the literature will look at their biological mechanisms and implications for a variety of health problems. Table 1 summarizes the key omega‐3s, as well as their sources, features and mechanisms.

**Table 1:** Various omega‐3s with their specific features, source and mechanisms

Name	Chemical structure	Mechanisms	Features	Source	References
ALA	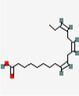	Contributes to anti‐inflammatory pathways. Acts as a precursor to EPA and DHA; however, the conversion rates are low. ALA supports proper cellular activity and communication by preserving the structural integrity of cell membranes	Exhibits anti‐inflammatory characteristics Enhances cognitive performance and may lower the incidence of neurodegenerative illnesses Cardioprotective and maintains skin health	Walnuts, flaxseed, chia seeds	24, 25, 26, 27
Docosahexaenoic acid (DHA)	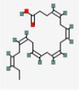	Improves neurotransmitter function and neurogenesis. Preserves and restores neuronal membrane function	Supports cognitive performance Key element of retinal tissue necessary for preservation Reduces the risk of neurodegenerative diseases	Fatty fish and Fatty fish, algae oil	28, 29, 30, 31
EPA	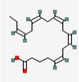	EPA contributes to the formation of anti‐inflammatory eicosanoids. Lowers blood pressure, lipid levels and improves heart health in general. EPA helps preserve the hydration and integrity of the skin	Boosts mood and can help ease anxiety and depression Potent anti‐inflammatory action Improves skin health	Fish oil and fatty fish (mackerel, salmon)	32, 33, 34, 35, 36, 37, 38
Stearidonic acid (SDA)	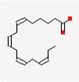	Stearidonic acid can be turned into EPA in the body. It may help to maintain the status of omega‐3s	Improves skin health Possibly enhances mood and cognitive abilities Acts as a cardioprotective	Found in certain plant oils like echium oils, hemp seeds	38, 39, 40, 41, 42
Eicosatetraenoic Acid (ETA)	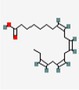	Reduces the synthesis of pro‐inflammatory cytokines and eicosanoids, hence reducing inflammation in the body	Lowers risk of psoriasis	Fish oil	43, 44, 45
Docosapentaenoic acid (DPA)	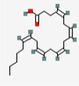	DPA facilitates the synthesis of the more active omega‐3s by acting as a metabolic intermediary Improves lipid profiles to promote heart health	Lowers the risk of heart Anti‐inflammatory effects Acts as a metabolic precursor	Fish and algae	46, 47, 48

## OMEGA‐3S AS FUNCTIONAL FOODS

Omega‐3s comprise a functional food that contributes significantly to a number of physiological functions, going beyond simple nourishment.^24^, ^49^, ^50^ Supplementation with omega‐3s has drawn great attention recently.^51^ They are frequently added to functional food items, such as dairy products,^52^ nutritional supplements^53^ and fortified eggs,^54^ so that customers can readily increase their consumption.^53^ Because of their adaptability, omega‐3s are a crucial component of various dietary patterns that satisfy a variety of food preferences, including pescatarian and vegan meals.^55^, ^56^ People can enjoy a range of flavors and textures at the same time as improving the nutritional profile of their diets by incorporating omega‐3‐rich foods. To increase the nutritional value of foods, a number of techniques have been developed to integrate omega‐3s. Manufacturers frequently use various omega‐3s rich dietary contents in their products, such as dairy products and snacks, that raise the omega‐3 composition in their products.^57^, ^58^ Processed foods frequently emulsify omega‐3 oils to ensure their seamless integration with other components. This process enhances the texture of the end product and preserves stability. Furthermore, certain foods are made with constituents that are inherently high in omega‐3s.^59^ Food grains, granola bars and baked products may incorporate these ingredients. However, the incorporation of omega‐3s into functional foods is a challenging task because of their low solubility in water and propensity to oxidation, which can impact their bioavailability as well as stability in dietary products.^40^, ^60^, ^61^ Enriching dietary products with polyunsaturated fatty acids (PUFA) markedly enhances their vulnerability to oxidation, resulting in quality degradation, undesirable flavours and certain health hazards.^4^, ^62^ Lipid oxidation poses a significant problem in the incorporation of PUFAs into meals, mostly as a result of their numerous reactive double bonds. PUFAs, chiefly EPA and DHA, are extremely prone to oxidation throughout handling and storage, resulting in the formation of harmful substances.^62^, ^63^, ^64^ Oxidation results in rancidity, diminished nutritional value and decreased shelf life. In animal products, it may also affect palatability and customer acceptance.^65^, ^66^, ^67^ Oxidized PUFA compounds can be cytotoxic, genotoxic, pro‐inflammatory and potentially cocarcinogenic, particularly when ingested in significant quantities or over extended durations.^68^, ^69^, ^70^, ^71^ Improved encapsulation technologies, including microencapsulation and nanoemulsions, have been developed to overcome these obstacles. These technologies enable the effective incorporation of omega‐3s into a diverse array of dietary supplements by preserving them from oxidation and improving their bioavailability and stability.^40^, ^72^

Omega‐3‐enriched foods were developed because of consumer demand with respect to a nutritious diet. These products provide a working solution to the problem of the inadequate consumption of these fatty acids in contemporary diets.^73^, ^74^ In general, omega‐3s are a crucial component of functional foods. These acids confer several health benefits and are essential for preventing diseases and health maintenance. As consumers become more health conscious and more concerned about preventing nutrition‐related illnesses, there is a growing demand among consumers for omega‐3s fortified functional foods.^75^, ^76^ However, a study revealed that most consumers obtain omega‐3s from natural sources or supplements, whereas only few consumers select foods fortified with omega‐3s.^73^ Consequently, this suggests that there is a potential commercial opportunity for the development of omega‐3s fortified functional foods that are more acceptable.^77^

## BIOAVAILABILITY

Omega‐3s are important nutrients, particularly long‐chain polyunsaturated fatty acids such as DHA and EPA.^1^, ^78^ EPA and DHA are vital nutrients that have many positive effects on health. Bioavailability differs greatly depending on their chemical structure and the way they are consumed. Triacylglycerols (TAGs), phospholipids (PLs), free fatty acids (FFAs) and ethyl esters (EEs) are some of the chemical forms of omega‐3s. Typically, FFAs have a higher bioavailability than EEs, whereas TAGs and PLs have different bioavailability depending on the study.^79^ Monoacylglycerols (MAGs) are lipid molecules consisting of a single fatty acid sequence linked to a glycerol backbone, usually formed through the digestion of dietary TGs. They help as significant intermediates in lipid metabolism and are gradually applied as carriers for bioactive fatty acids, such as omega‐3s.^80^, ^81^ Following a low‐fat diet, the pre‐digested MAG form of omega‐3s has demonstrated noticeably higher absorption than the ethyl ester form, perhaps making it a more efficacious therapeutic alternative.^80^ Dietary lipids may have an impact on how well omega‐3s are absorbed. For example, when omega‐3 ethyl esters are taken with fat diets, their bioavailability is increased.^82^ Many factors affect the long‐chain bioavailability of omega‐3s. In addition to the type of chemical bond, the uptake of omega‐3s are influenced by the presence of other components and the concurrent consumption of food, particularly its fat content. For example, calcium ions have the capacity to combine with FFA to create a complex, which lowers their availability.^79^ Encapsulation strategies may enhance the bioavailability and stability of omega‐3s by preventing oxidation and increasing utilization in the epithelium of the intestinal tract.^83^ According to experimental evidence, omega‐3s from fish are more effectively absorbed into plasma lipids than when given as capsules, and increases in plasma concentrations of EPA and DHA given as capsules are linearly associated with their intakes.^84^ In conclusion, the chemical form, dietary context and methods used to stabilize and transport omega‐3s all influence their bioavailability, although they provide an extensive range of health benefits.

## METABOLISM

The body uses omega‐3s for a number of metabolic functions that are essential for normal body physiologic functions. Bile salts emulsify omega‐3s in the intestine and pancreatic lipases break them, converting them into monoglycerides and free fatty acids that can be absorbed. After being absorbed, omega‐3s break down into chylomicrons and are re‐esterified into TGs.^85^, ^86^ Additionally, chylomicrons move fatty acids to different tissues by entering the lymphatic system and subsequently the bloodstream. Certain transport proteins allow cells to absorb omega‐3s. Transport proteins embedded in cell membranes, such as fatty acid transport proteins (FATPs) and fatty acid translocase (FAT/CD36), are mostly responsible for the cellular intake of omega‐3s.^87^, ^88^ These proteins allow for the effective entry of omega‐3s and can also pass passively through the lipid bilayer in their free state. After entering the cell, omega‐3s can be re‐esterified into TGs or incorporated into phospholipids, which are critical to sustaining cell membrane structure and function.^12^, ^89^ This absorption process is critical for gaining the health benefits of omega‐3s, including their role in energy production. Omega‐3s can undergo β‐oxidation in mitochondria, converting them into acetyl‐CoA that can be used in the Krebs cycle for energy production.^90^, ^91^

**Figure 1:**
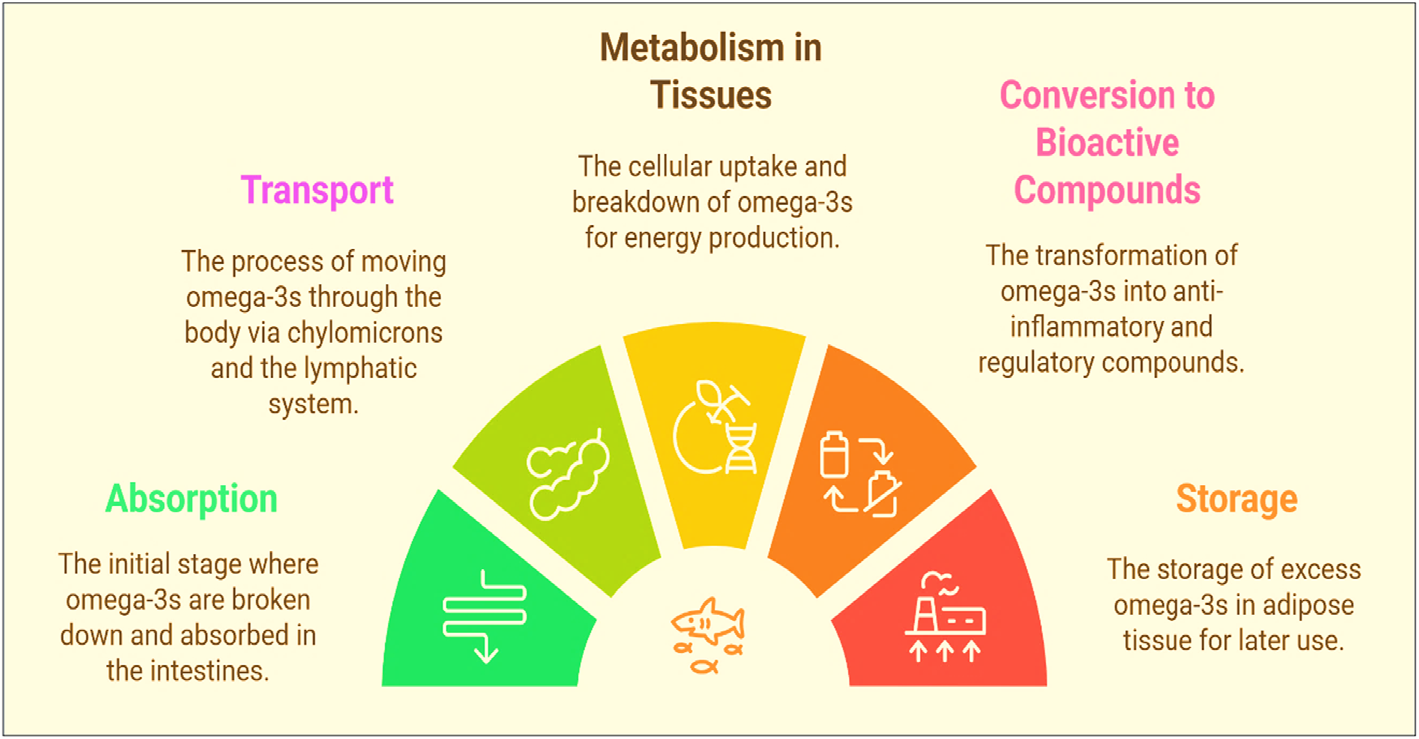
Various steps involved in omega‐3s metabolism.

In many tissues, notably the brain and immune cells, omega‐3s, especially EPA and DHA, are converted into bioactive substances by enzymatic mechanisms. Eicosanoids such as prostaglandins and leukotrienes are produced from EPA. Additionally, certain omega‐3s contribute to the production of endocannabinoids, which regulate inflammation and pain.^92^, ^93^ On the other hand, excess omega‐3s can be deposited as TGs in adipose tissue. When exercising or fasting, they might be released to provide energy. When all factors are considered, the metabolism of omega‐3s is a multifaceted process that involves several organs and enzymes and produces a range of physiologically active substances that support human health and well‐being (Fig. 1).

## MECHANISMS OF ACTION

ALA produces potential role through various mechanisms.^94^ It can be integrated into cell membranes, modifying their fluidity and displacing arachidonic acid (AA), which decreases the existing substrate for the production of pro‐inflammatory eicosanoid. Lipoxygenase (LOX), cyclooxygenase (COX) and cytochrome P450 (CYP450) pathways also break down ALA into oxylipins. These oxylipins directly reduce inflammation and change the way the immune system functions. For example, they lower the levels of inflammatory cytokines and improve lipid profiles and act as cardioprotectives.^95^, ^96^ This increases the activity of the Nrf2/HO‐1 pathway, which lowers the levels of reactive oxygen species (ROS) and oxidative stress in brain tissues. These modes of action protect against neurodegenerative disorders and support mental health.^97^, ^98^, ^99^ In addition, stimulation of nuclear factor‐kappa B (NF‐κB) and mitogen‐activated protein kinase is prevented, which are significant for controlling the expression of inflammatory genes. This decreases the production of the pro‐inflammatory substances tunor necrosis factor (TNF)‐α, interleukin (IL)‐1β, inducible COX‐2 and inducible nitric oxide synthase (iNOS). Such a mechanism has the potential to exhibit anti‐inflammatory properties.^100^, ^101^

EPA alleviates oxidative stress by lowering ROS and improving antioxidant action, such as NRF2 upregulation. It also modulates gene expression via microRNA in adipose tissue, leading to decreased inflammation and enhanced insulin sensitivity, as well as improved metabolic homeostasis. These mechanisms are chiefly significant in obese and diabetic people, where persistent inflammation and oxidative stress promote disease progress.^102^, ^103^, ^104^ Moreover, EPA competes with AA for incorporation into cell membranes and for metabolism by COX and LOX enzymes. This competition leads to the production of a lesser amount of inflammatory eicosanoids and the generation of specialized pro‐resolving mediators, such as resolvin E1, which actively resolve inflammation. EPA has the potential to reduce the pro‐inflammatory cytokines expression and chemokines, such as TNF‐α IL‐1β and IL‐6.^105^, ^106^ Additionally, it has the ability to inhibit the activation of NF‐κB, which is a crucial regulator of the expression of inflammatory genes. Through altering the fluidity and structure of the membrane, it makes atherosclerotic plaques more stable and decreases the possibility of rupture. It accomplishes this by lowering the levels of TGs in the plasma by decreasing the formation of very‐low‐density lipoprotein (VLDL) in the liver and increasing the clearance of lipoproteins that are rich in TGs. PA likewise increases endothelial function by raising bioavailability of nitric oxide (NO), which promotes vasodilation.

DHA is highly concentrated in the brain, cell membrane and retina. It is essential for usual development and function in life.^107^ DHA enhances cell membrane fluidity and associates with membrane protein function, which is essential for effective cell signaling, particularly in neurons. DHA regulates signaling pathways by triggering several receptors and kinases, hence enhancing neuronal survival, synaptic development and neurogenesis. It also promotes the production of phosphatidylserine, a membrane lipid that increases the activation of essential kinases implicated in neuronal development and protection.^108^, ^109^ DHA is enzymatically transformed into bioactive lipid mediators, comprising resolvins, protectins and neuroprotectins, which have potent anti‐inflammatory and neuroprotective properties. These metabolites exhibit anti‐inflammatory action and protection against oxidative stress and promote tissue repair. DHA promotes the Nrf2/ARE antioxidant pathway, enhancing cellular antioxidant protection and preserving oxidative homeostasis.^109^, ^110^, ^111^, ^112^ DHA modulates mitochondrial activity, enhances mitochondrial biogenesis and facilitates fatty acid oxidation, which lowers lipid accumulation and helps energy metabolism. It also regulates the expression of gene linked to inflammation and lipid metabolism, revealing multifaceted mechanisms against cardiac, obesity and neurodegenerative disorders.^111^, ^113^, ^114^

## HUMAN HEALTH

Omega‐3s are essential for maintaining overall health and well‐being. They are mostly found in nuts, seeds and certain types of seafood, being required for a variety of bodily functions, including heart and brain health and inflammation reduction. This review explores some of the most significant health benefits associated with these excellent fatty acids.

### Cardioprotection

ALA, EPA and DHA are termed omega‐3s. They have a beneficial cardioprotective effect, making them a valuable part of a healthy diet and lifestyle. Different studies have reported the vital role of omega‐3s as having anti‐inflammatory, vasodilating, antiarrhythmic, antihypertensive and anticoagulant effects. They also play a critical role in decreasing triacylglycerol levels.^115^, ^116^, ^117^ Below, a summary is provided of their functions under particular circumstances.

#### Coronary heart disease (CHD)

EPA and DHA, found mainly in seafood (especially fatty fish), are associated with cardioprotective properties as a result of the modulation of blood lipids, blood pressure, heart rate, aggregation of platelets, functions of endothelium and inflammation.^118^ The incidence of CHD is increasing worldwide with varying etiological factors. Modification of the diet can prevent and reduce the prevalence of CHD risk. Epidemiological and randomized controlled studies have reported that consumption of marine omega‐3s (EPA and DHA) reduces mortality and morbidity as a result of CHD.^119^ The therapeutic effect of EPA and DHA is the key mechanism responsible for lowering CHD and mortality in patients with the disease. Because they produce altered eicosanoid mediators that reduce platelet aggregation, these fatty acids have anti‐thrombotic, antioxidant and anti‐inflammatory properties that stabilize atherosclerotic plaques and prevent their rupture. They also lower TG levels by reducing the production of hepatic TGs and increasing the clearance of plasma TGs.^120^, ^121^

Del Gobbo *et al*.^122^ pooled 19 cohort studies showing that consumption of higher concentrations of any of the four individual omega‐3s, ALA, EPA, DHA and docosapentaenoic acid (DPA), is associated with a reduction of CHD. The omega‐3s are an important component of the membrane of the platelet phospholipid; hence, they play a vital role as antiplatelets.^113^ Additionally, they also alter the cell structure and signaling by changing the lipid configuration within the cell membrane.^123^ EPA and DHA reduce CRP and pro‐inflammatory cytokines, resulting in increased arterial compliance and flow‐mediated dilation.^124^ The first report on the antithrombotic potential of omega‐3s was published by Innes *et al*.^118^ who observed the dietary pattern of Eskimos from Greenland. The incidence of cardiovascular disease was lower in the Eskimos because of the inclusion of fatty fish in their diet. Similar findings were also observed in a Japanese population who consumed one meal with fish every day, which accounted for approximately 900 mg of omega‐3s.^125^

Studies have reported that individuals who include fatty fish in their diet on a weekly basis have a much lower risk of CVD compared to those who do not consume fish in their diet.^126^, ^127^ The risk of CHD can be prevented by maintaining an effective level of blood concentration of omega‐3s, which can be attained by an intake of 1 g of EPA along with DHA every day. Hence, the American Heart Association recommends that an adult consumes two meals with fatty fish per week to prevent primary cardiovascular disease complications.^128^ Mozaffarian *et al*.^129^ have reported a lower incidence of heart failure in older adults with a higher plasma concentration of omega‐3s. Several factors are responsible for the variation in the beneficial effect of omega‐3s against CHD as reported in various clinical studies. This may be a result of the dose, duration of supplementation, EPA/DHA ratio, lack of measuring the effective blood level of omega‐3s, co‐morbidities (hypertension, stroke and diabetes), interaction between drugs (statin, aspirin, etc.) and bioavailability of omega‐3s.^129^

The Food and Drug Administration, Canada and the European Union (EU) have approved icosapent ethyl (IPE) as a purified EPA. The use of IPE is recommended to reduce the risk of cardiovascular disease in a patient with atherosclerosis.^130^ Consumption of higher doses of IPE can reduce elevated TGs, which can benefit CHD patients.^131^ Therefore, the consumption of highly purified EPA can prevent complications in atherosclerosis patients with a risk of cardiovascular disease. The prevention and treatment of CHD could be achieved by the inclusion of omega‐3s in the diet.^132^ Their cardiovascular protective, lipid‐modifying and anti‐inflammatory qualities help to lower the risk factors for CHD. It is advised to regularly eat foods high in omega‐3s, such as fatty fish, as part of a diet that promotes heart health.

#### Hypertension

Hypertension is a major modifiable risk factor for cardiovascular diseases, characterized by impaired vasodilation because of multiple dysfunctions of vasodilatory mechanisms. Primary hypertension (90–95%) is associated with lifestyle and genetic factors. Hypertension increases the incidence of cardiovascular death and the risk of cerebrovascular, coronary, peripheral vascular disease, heart failure and chronic kidney disease.^133^ Primary hypertension is a complex pathophysiological condition characterized at the peripheral vascular level by an imbalance between vasoconstriction and vasodilatation. Lifestyle change is highly recommended as a first step of treatment for patients with hypertension^134^ because of its ability to reduce blood pressure. Dietary interventions play an important role in weight loss by restricting calories. DHA and EPA supplementation helps to regulate and reduce blood pressure and vasodilation.^135^ Studies have reported that elevated levels of omega‐3s in the blood may be associated with a decreased risk of hypertension.^136^ Various subsequent long‐term cohort studies and short‐term randomized controlled studies also confirm that higher intake of omega‐3s is associated with lower blood pressure in hypertensive patients.^137^

Zhang *et al*. evaluated the relationship between omega‐3s and antihypertensive efficacy in a randomized controlled study, reporting an optimal dose of 2 g per day (−2.61/−1.64 mmHg) to 3 g per day (−2.61/−1.80 mmHg) is required for lowering blood pressure, with an especially strong and linear dose–response relationship in the aged, hypertensive and hyperlipidemic populations.^135^ Omega‐3s decrease resting systolic and diastolic blood pressure by the incorporation of EPA and DHA into the phospholipid membrane, thereby increasing systemic arterial compliance and decreasing resting systolic and diastolic blood pressure.^138^, ^139^ Various clinical studies and meta‐analyses report the beneficial effects of omega‐3s in regulating blood pressure in both hypertensive and normotensive individuals through the regulation of the vascular tone mediated by both endothelium‐dependent and independent mechanisms.^140^

Consuming omega‐3s lowers blood pressure through several mechanisms such as vasodilation, improving flexibility of the blood vessels, reducing inflammation, regulating heart rate with an antiarrhythmic effect and improving endothelial function.^141^, ^142^ The endothelium plays a crucial role in regulating vascular health by releasing vasoactive substances such as NO, endothelium‐derived hyperpolarizing factor (EDHF), and prostacyclin (PG12), which maintains homeostasis. Endothelial cells act as a barrier between the blood and interstitial space; they also express molecules of adhesion, such as intercellular adhesion molecule 1 (ICAM‐1) and vascular cell adhesion molecule 1 (VCAM‐1), which are involved in mediating the inflammatory process associated with atherosclerosis.^143^ The omega‐3s inhibit inflammation by altering the endothelial cell membranes lining the arterial lumen by suppressing the expression of adhesion molecules and producing anti‐inflammatory eicosanoids.^120^, ^144^, ^145^ Nitric oxide mediates the dilation of vascular smooth muscle cells by increasing the production of cyclic guanosine monophosphate (cGMP), which results in the activation of cGMP‐dependent protein kinase. The release of NO from the endothelium is necessary for the vasodilatory response. As it plays a crucial role in controlling blood pressure.^146^

Previous investigations have demonstrated that patients with essential hypertension have compromised NO‐mediated coronary artery vasodilation. Studies have also reported the role of omega‐3s in enhancing the production of NO from the endothelial cells and improving endothelial function.^147^, ^148^, ^149^ Increased superoxide generation and decreased NO bioavailability have been identified as the mechanisms underlying endothelial dysfunction in hypertension. The endothelium's capacity to shield the vasculature against oxidative stress, inflammation, thrombosis and atherosclerosis is compromised in the absence of NO. Reduced bioavailability of NO and an increase in the production of superoxide are attributed to endothelial dysfunction in hypertension. Absence of NO results in the loss of the endothelium's ability to protect the vasculature against oxidative stress, inflammation, thrombosis and atherosclerosis.^150^ Hence, many studies have reported the beneficial effect of the consumption of omega‐3s by hypertensive individuals, which is a major risk factor for stroke, cardiovascular disease, and kidney disease.

#### Hypertriglyceridemia

TGs, cholesterol and phospholipids are the components of lipids in human blood. TG is a vital biomarker of blood lipids, required for the transportation and storage of energy. The development of atherosclerosis, cardiovascular disease with a risk of ischemic stroke, peripheral arterial disease and myocardial infarction is caused by the elevation of TGs.^151^, ^152^ Higher levels of plasma TG are caused as a result of excess TG‐rich lipoproteins, such as VLDLs and intermediate‐density lipoproteins (or VLDL remnants), chylomicrons or chylomicron remnants. Omega‐3s lower the level of TG in the serum by reducing the synthesis of TG, decreasing the incorporation of TG into VLDL, lowering TG secretion and enhancing the clearance of TG from VLDL particles, in turn lowering plasma TG cholesterol levels; hence, the probability of atherogenesis is reduced.^153^ It is widely accepted that omega‐3s enhance the degradation of fatty acids and accelerate the removal of TGs from the plasma, which lowers serum levels of TG in part by reducing the hepatic synthesis of VLDL.^129^, ^154^

Omega‐3s accelerate the beta‐oxidation of fatty acids (the biological pathway that breaks down fat into energy) and lipoprotein‐lipase expression, which influences the suppression of lipogenic gene expression, thereby influencing the accumulation of total body lipid.^153^, ^155^, ^156^ Hence, the level of TGs is decreased in the body because of an increase in the rate of beta‐oxidation, which acts specifically on carnitine acetyltransferase 1 (CAT1) and acetyl‐CoA carboxylase.^153^, ^155^ Carnitine acetyltransferase modifies fatty acid substrates to enter the inner mitochondrial membrane via carnitine‐acylcarnitine translocation; subsequently, they are converted to acyl‐CoA, a precursor substrate to acetyl‐CoA, and is utilized in various metabolic pathways to generate ATP. Additionally, EPA also indirectly increases beta‐oxidation by slowing feedback inhibition. EPA inhibits acetyl‐CoA carboxylase, which is the enzyme that catalyzes the synthesis of malonyl‐CoA, a strong inhibitor of CAT1. Decreased production of malonyl‐CoA will increase CAT1 activity, thereby using maximum TG for beta‐oxidation. Studies show that omega‐3s also decrease the sensitivity of CAT1 to malonyl‐CoA.^155^

**Figure 2:**
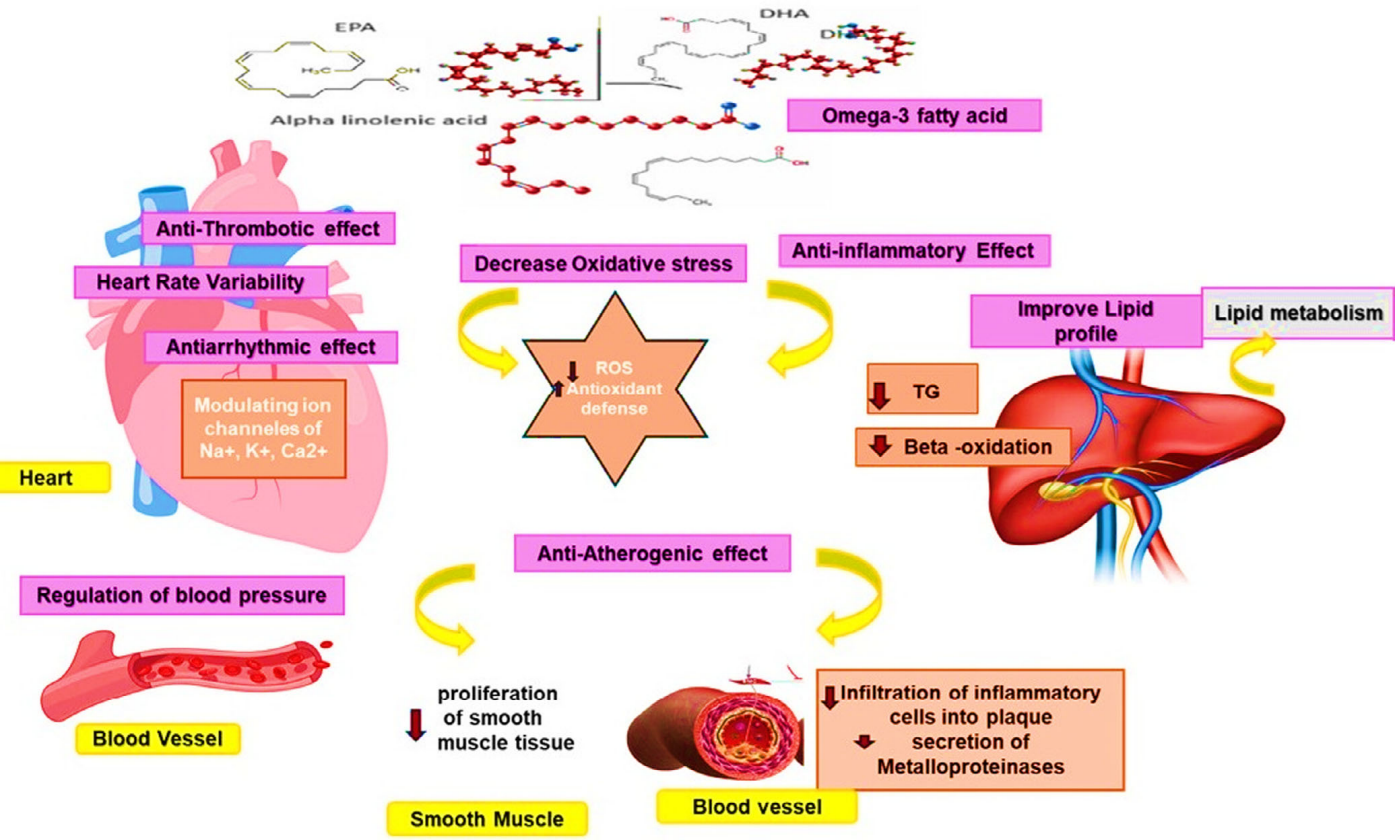
A multidimensional approach to the cardioprotective effects of omega‐3 fatty.

Omega‐3s influence the accumulation of total body lipid by reducing higher levels of TG. Numerous studies have reported that prolonged use of omega‐3s for more than 6 weeks can increase the body's metabolic rate and decrease total body fat.^157^, ^158^ Omega‐3s can also reduce the liver and plasma TG through the generation of *N*‐acyl taurine. Taurine is an organic compound that is derived from cysteine and, when it is conjugated to bile acids, facilitates the emulsification and absorption of fats.^159^ Omega‐3s increase fat oxidation and energy needs by changing the body composition, which is considered to be another mechanism by which omega‐3s help lower the TG levels in the blood.^160^ Serum TG level has long been recognized as a risk factor for CVD because of its role in the release of coagulation factors, pro‐inflammatory cytokines and impairment of fibrinolysis.^161^ Four grams of omega‐3s per day can significantly lower the TG level and thus can be considered a therapeutic option for those with elevated TG levels.^162^ However, the effect on high‐density lipoprotein‐cholesterol (HDL‐C) or low‐density lipoprotein‐cholesterol (LDL‐C) is uncertain concerning the EPA/DHA ratio. A study has revealed that omega‐3s have a lower impact on HDL‐C and LDL‐C concentrations.^163^ Thus, the effect of omega‐3s in reducing the risk of atherosclerotic cardiovascular disease (ASCVD) is partially associated with lowering the TG levels.^164^ Omega‐3s decrease serum TG levels by reducing TG synthesis, TG incorporation into VLDL and TG secretion, as well as enhancing TG clearance from VLDL particles.^153^ The cardioprotective mode of action is depicted in Fig. 2.

### Cancer

**Figure 3:**
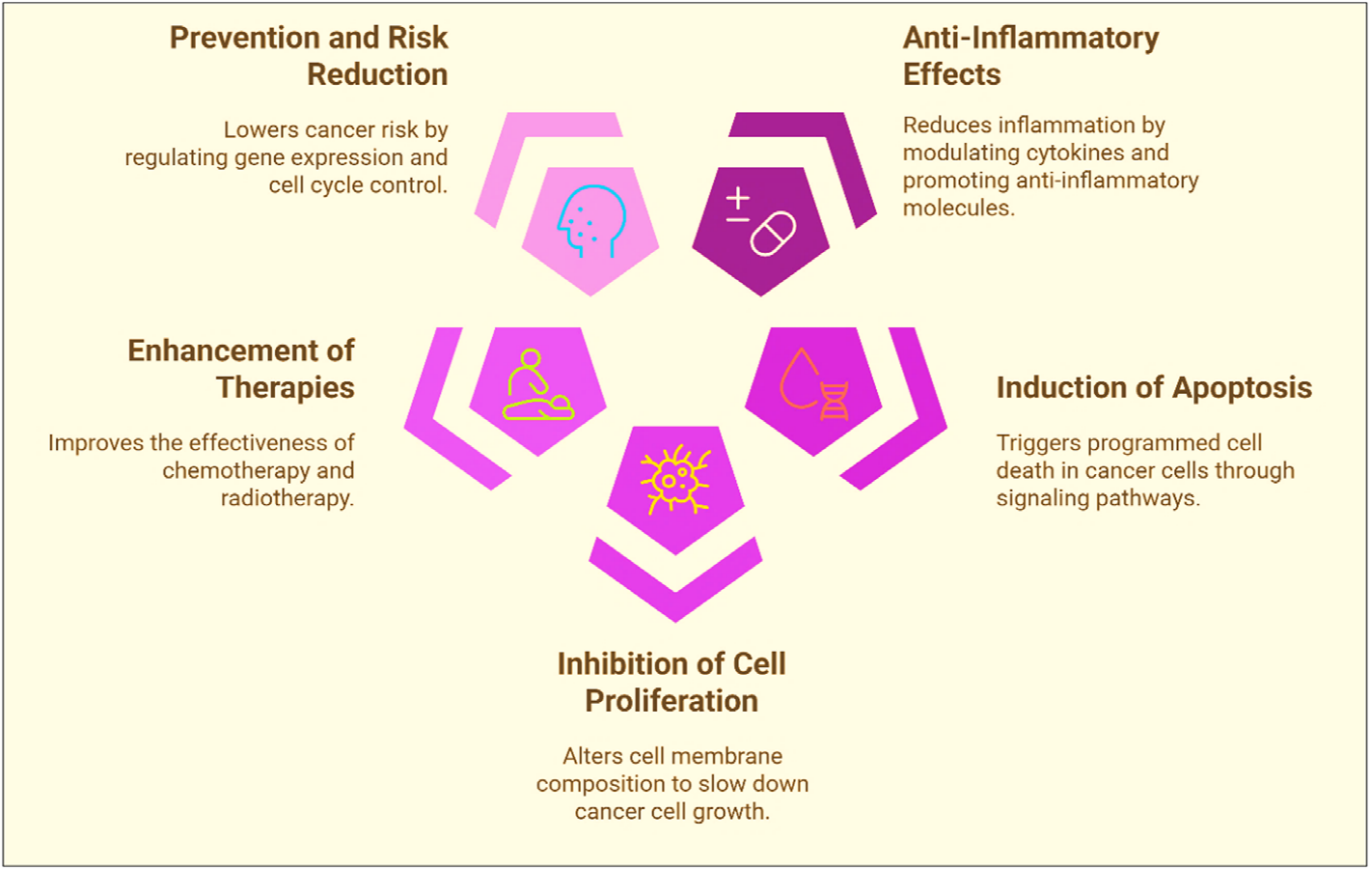
Multifaceted mechanisms exhibited by omega‐3s against cancer.

The term ‘cancer’ refers to a group of disorders that are characterized by the uncontrolled growth and spread of cells.^165^ The diet for a cancer patient should include a variety of nutrients.^166^ Several clinical and epidemiological studies have reported omega‐3s as the most effective nutrient required for maintaining health in these individuals because they have antioxidant, antitumor, anticarcinogenic and neuroprotective properties, which help the cancer patients to reduce their various physical challenges.^167^, ^168^, ^169^ Antioxidant properties of omega‐3s (DHA and EPA) aid with increasing antioxidant enzymes by removing the reactive oxygen species and reducing the oxidative stress; they also provide effective support for treatment in cancer patients through suppression of COX‐2 expression and giving resistance to mitosis, resulting in a controlled growth by restoring the apoptotic pathway.^170^ Various beneficial effects of omega‐3s in the prevention and management of cancer are shown in Fig. 3 and Table 2. These fatty acids suppress the growth of tumor cells, decrease the cancer‐induced pain, act as a crucial component in the maintenance of major depression disorder (MDD), prevent anorexia cachexia syndrome, and prevent paraneoplastic syndrome of cancer with multiple clinical complications such as neurological, hematological, dermatological, rheumatological and endocrinological complications.^180^ Omega‐3s play an important role in the management of numerous types of cancers; this review focuses specifically on lung, liver and colon cancer because the incidence of these cancers is becoming more common in both developed and developing countries. An outline of their effects on various cancers is provided below.

**Table 2:** Summary of the function of omega‐3s in cancer, including their possible mechanisms of action

Omega‐3s	Types of cancer	Mechanisms of action	Potential benefits	References
ALA	Colorectal, breast	Displays anti‐inflammatory properties Functions as an antioxidant. Alters the metabolism of fats Affects the expression of genes linked to apoptosis	Potentially lowers cancer risk and improves overall health	171, 172
DHA	Breast, prostate, pancreatic	Boosts the immunological system Decreases inflammation Modifies the composition of the cell membrane. Modifies the survival of cancer cell signaling pathway	Supports immune function and may enhance chemotherapy efficacy	173, 174, 175, 176
EPA	Breast, colorectal, prostate	May reduce tumor growth and improve treatment outcomes	Alters inflammation Suppresses angiogenesis Promotes apoptosis Modulates progression of expression of cancer‐causing gene	177, 178, 179

#### Lung cancer

The most common cause of cancer‐related deaths worldwide is lung cancer. It is differentiated into two types: non‐small cell lung cancer (NSCLC) and small cell lung cancer (SCLC).^181^ NSCLC accounts for approximately 85% of all lung cancer cases and is generally less aggressive than SCLC. Treatment options for NSCLC often include surgery, chemotherapy and targeted therapies, depending on the stage of the disease and the patient's overall health, accounts for 85%, further categorized as adenocarcinoma, squamous cell carcinoma and large cell carcinoma. NSCLC is the most common type of lung cancer; squamous cell carcinoma and large cell carcinoma metastasize easily, resulting in difficulties for treatment.^182^, ^183^, ^184^ SCLC is more lethal because it proliferates more rapidly. Currently, chemotherapy is used as the most significant treatment for lung cancer, as it has shown improvement in the survival rate. However, the survival rate of SCLC through chemotherapy is low because of its recurrence. Alternative treatments include radiation therapy and immunotherapy, which can be safely administered along with chemotherapy to improve its effectiveness and improve survival rates against recurring cancer.^185^, ^186^

There is evidence that the causative factor for cancer is a change in lifestyle. Lifestyle choices can be potentially modifiable, such as diet, which can help in the prevention of different types of cancer.^187^ Diets containing anti‐inflammatory components such as omega‐3s play a vital role because inflammation is the major pathogenesis in cancer. EPA and DHA reduce the risk of cancer because they inhibit phorbol 12‐tetra decanoate 13‐acetate (TPA) and epidermal growth factor (EGF), which induces the growth of tumors.^188^ Omega‐3s are immune nutrients that are frequently used in the nutritional therapy of cancer patients; they are essential for cellular signalling, structure and membrane fluidity. They have anti‐inflammatory properties and help in addressing the cause of inflammation. A study conducted by Patchen *et al*. (2019) on smokers has reported that consumption of omega‐3s (DPA, DHA and EPA) was positively correlated with higher levels of forced expiratory volume (FEV) and higher forced vital capacity (FVC). However, patients with lung cancer or those who have had lung cancer in the previous years can benefit from these positive effects of omega‐3s.^189^

**Figure 4:**
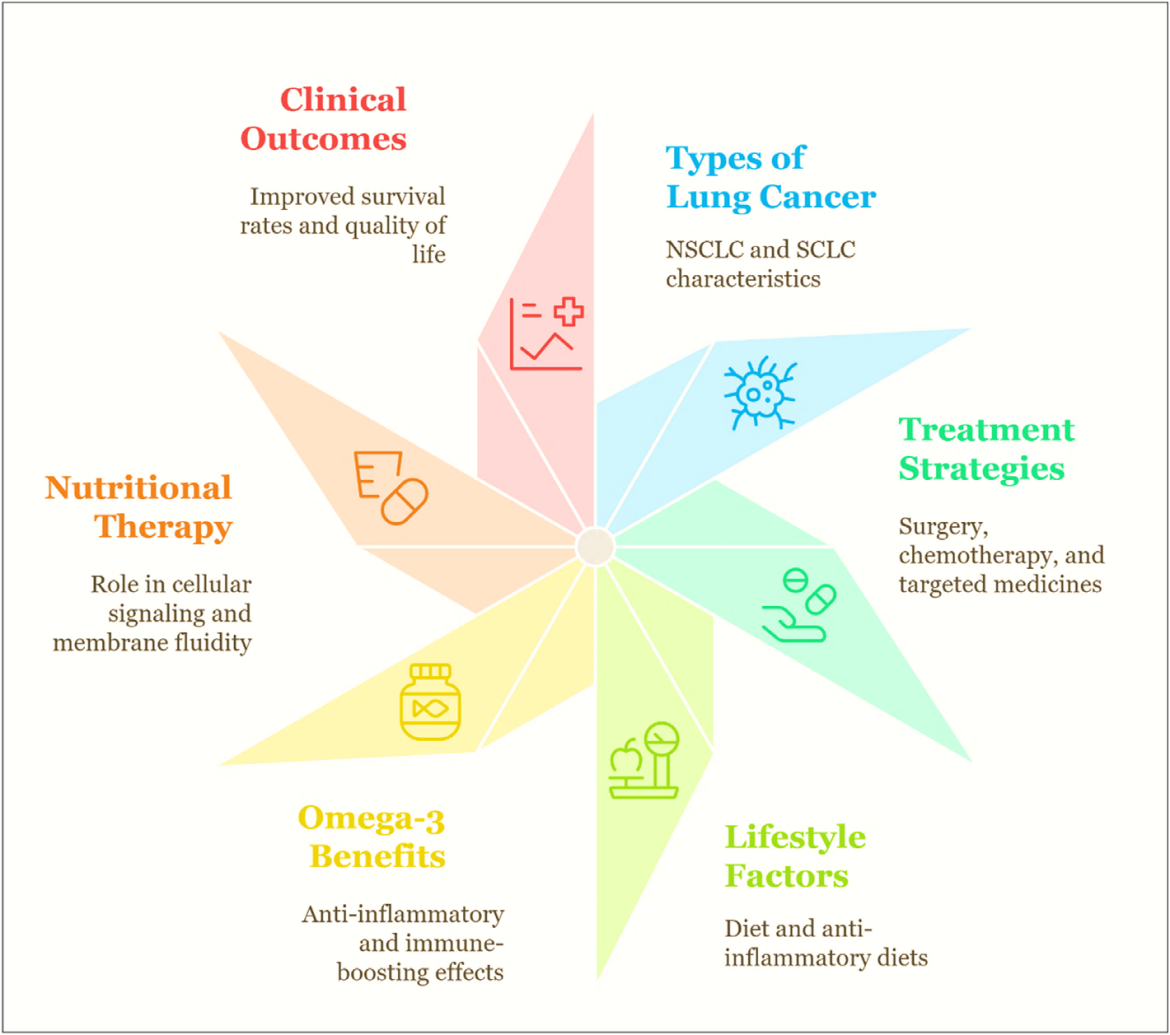
Showing the type of lung cancer (NSCLC and SCLC) with their corresponding treatment modalities.

Chemotherapy causes esophagitis, nausea and other side effects that impair the nutritional status and overall well‐being of a patient. Malnutrition is prevalent in 45–69% of patients with lung cancer, which is associated with poor responses to chemotherapy and other cancer treatments.^190^ Various studies have reported that supplementation with omega‐3s increases the muscle mass of the skeleton, regulates inflammatory responses, lowers the risk of gastrointestinal reactions, reduces anorexia, and enhances patients' tolerance to chemotherapy, radiation and surgery with NSCLC, thus increasing their lifespan.^191^ Numerous studies have extensively studied and demonstrated the immunomodulatory and anti‐inflammatory properties of omega‐3s in cancer patients.^192^, ^193^ Additionally, patients with stage II‐III NSCLC undergoing postoperative chemotherapy have shown lower chronic inflammatory responses and improved in the nutritional status after supplementation with omega‐3s (Fig. 4).^194^

#### Liver cancer

Globally, liver cancer is ranked fourth among the most prevalent types of cancer.^195^, ^196^ The most common primary liver cancer is hepatocellular carcinoma (HCC), accounting for 85–90%, representing a significant obstacle and impact on medical systems in many countries worldwide.^197^, ^198^ Lack of early diagnosis is a characteristic feature of HCC. Therefore, current studies focus on understanding and elucidating the signalling pathways involved in the progression of HCC as a therapeutic target.^199^, ^200^ However, the process of progression, migration and suppression of apoptosis in HCC is a result of the involvement of signal transducer and activator of transcription 3 (STAT3).^197^ Studies have found that natural products such as omega‐3s are capable of addressing the STAT3 signaling pathway and suppressing malignancy and its proliferation.^201^ Moreover, some studies using cultured cancer cells have demonstrated that supplementation with omega‐3s induced apoptosis and reduced proliferation of cells.^202^ Omega‐3s inhibit signal transduction and down‐regulate genes responsible for elevating signals of carcinogenesis.^203^ Furthermore, they also inhibit the growth of hepatobiliary tumor cells by blocking beta‐catenin and COX‐2 signaling pathways.^204^ Numerous observational studies conducted on the human population have reported retarded growth and metastasis of primary tumors of breast, liver, colon and prostate cancer by the consumption of higher amounts of dietary omega‐3s or fish.^205^, ^206^, ^207^ Omega‐3s are involved in various cellular and molecular processes. They are an important component of the circulating lipid pools and are transformed into bioactive metabolites that are involved in signal transmission. Moreover, they can also alter both the physical and chemical properties of cell membranes and modify the proteins and membrane channels.^208^, ^209^

A study conducted by Jiang *et al*.^210^ reported that habitual use of fish oil supplements (a source of omega‐3s) significantly reduced the risk of liver cancer compared to individuals who did not include fish oil in their diet. An additional study by Ma *et al*.^211^ reports, in a study summarizing data from different cohort studies, that the intake of fish reduces the risk of liver cancer.

#### Colon cancer

Globally, the leading cause of cancer‐related deaths is colorectal cancer (CRC), also known as colon cancer.^212^ Colon cancer is an aggressive and life‐threatening malignancy that metastasizes in patients (50%), which results in significant challenges for survival and treatment.^213^ A diet rich in omega‐3s is an important part of the nutrition for cancer patients as a result of their beneficial effects, such as anti‐inflammatory properties, which improve the immunity of the body by modulating inflammation specifically in the colon's pro‐carcinogenic inflammatory state.^212^ Fatty acid metabolism regulates cell proliferation, metastasis, angiogenesis, immune suppression and drug resistance in patients with colon cancer.^213^ Anti‐inflammatory and anti‐CRC effects of EPA and DHA are also reported by many studies.^214^ They play an important role in multiple stages of CRC management, starting from the prevention of primary colon cancer until advanced metastasis.

The findings of several recent research on dietary status, inflammation and chemotherapy recovery are controversial. Omega‐3 supplementation has been demonstrated to lower CRP levels and shorten the duration of the systemic inflammatory response syndrome.^205^ Preclinical and epidemiological studies provide strong evidence of the anti‐colorectal cancer activity of omega‐3s. Dietary intake of omega‐3s reduces the risk of CRC by managing several stages of colon cancer from primary CRC prevention to tertiary prevention after treatment of colon cancer and advanced metastatic disease.^215^ Omega‐3s exhibit numerous anti‐CRC properties by regulating COX activity (inflammation), altering receptor function and cell membrane dynamics (impact cellular signaling), enhancing cellular oxidative stress (destruction of cancer cells), and producing new anti‐inflammatory lipid compounds such as resolvins, protectins and maresins, which are derived from EPA and DHA. These compounds aid in the resolution of inflammation and promote tissue repair.^216^

Lower consumption of dietary omega‐3s is associated with an increased incidence of cancers. EPA and DHA mainly affect cancer‐associated symptoms, such as postoperative complications, inflammation, cachexia, neuropathy and quality of life. Omega‐3s regulate these mechanisms by regulating protein turnover of skeletal muscle, inflammatory response and survival of neuron cells.^217^ The impact of enteral and parenteral supplementation with omega‐3s and other nutrients on clinical outcomes of CRC patients undergoing resection, such as weight, postoperative complications, infections and proinflammatory cytokine levels, was determined by Xie and Chang,^214^ who reported that the complication of hospital stay, infection, and plasma levels of IL‐6 and TNF‐α was reduced with omega‐3 enriched nutrition. Read *et al*.^218^ also found that giving omega‐3s to CRC patients before chemotherapy caused them to gain a lot of weight (about 2.5 kg on average). Even though they ate less protein and energy, the weight gain stayed after the chemotherapy started.^218^ Various studies have evaluated omega‐3 therapy as a complementary therapy to standard systemic treatment by examining various outcomes such as quality of life, tolerability to treatment and the systemic inflammatory response. There is reported evidence that the Glasgow Prognostic Score (GPS) is significantly reduced by supplementation with omega‐3 capsules in patients undergoing preoperative radiation therapy for locally advanced rectal cancer.^219^

### Central nervous system (CNS)

Omega‐3s are potential in neurological disorders because they may lower inflammatory conditions, promote neuronal health and enhance cognitive performance. Key points about their impact are outlined below.

#### Neurodegenerative diseases

##### Parkinson's disease

Parkinson's disease (PD) is another neurological disorder that affects the elderly. It is characterized by generalized movement slowing, tremors at rest and muscular rigidity. Later on, the patient develops loss of smell, drooling, sleep disturbances, constipation, extremity movements during sleep and mood disorder.^220^ Omega‐3s have been extensively studied for their anti‐inflammatory and neuroprotective properties. These fatty acids are an integral part of the nerve cell membrane, maintaining fluidity, reducing oxidative stress and modulating inflammation. These characteristics all help in controlling PD and other neurological disorders.^221^, ^222^ Omega‐3s help in reducing the production of proinflammatory cytokines, such as tumor necrosis factor‐alpha and some interleukins, and this helps in neuron protection in many neurodegenerative diseases.^223^ As stated earlier, the antioxidant action also helps with reducing oxidative stress and damage to brain cells. Oxidative stress is especially bad for dopaminergic neurons. Omega‐3s provide much help in PD by blocking free radicals.^224^ Furthermore, omega‐3s spare dopaminergic neurons from damage by protecting their sheets, and loss of this neuroplasticity results in damage to the brain and neurons. Loss of these neurons is considered to be the main pathology occurring in the substantia nigra in PD.^225^

According to clinical research, omega‐3s may be used as a treatment for PD, either as a stand‐alone intervention or in conjunction with already available pharmaceutical treatments. In PD patients, omega‐3 supplementation has been linked to improvements in depressed symptoms, suggesting potential advantages beyond the treatment of motor symptoms.^226^ Omega‐3s are also affordable and well‐tolerated, which makes them a beneficial choice for long‐term use.^225^, ^227^ Although preclinical and some clinical evidence is encouraging, more investigations are required to validate the effectiveness of omega‐3s in the treatment of PD. To determine the best dosages, lengths of treatment and long‐term impacts of omega‐3 supplementation in PD patients, extensive clinical trials are required.^225^, ^227^

##### Alzheimer's disease

**Figure 5:**
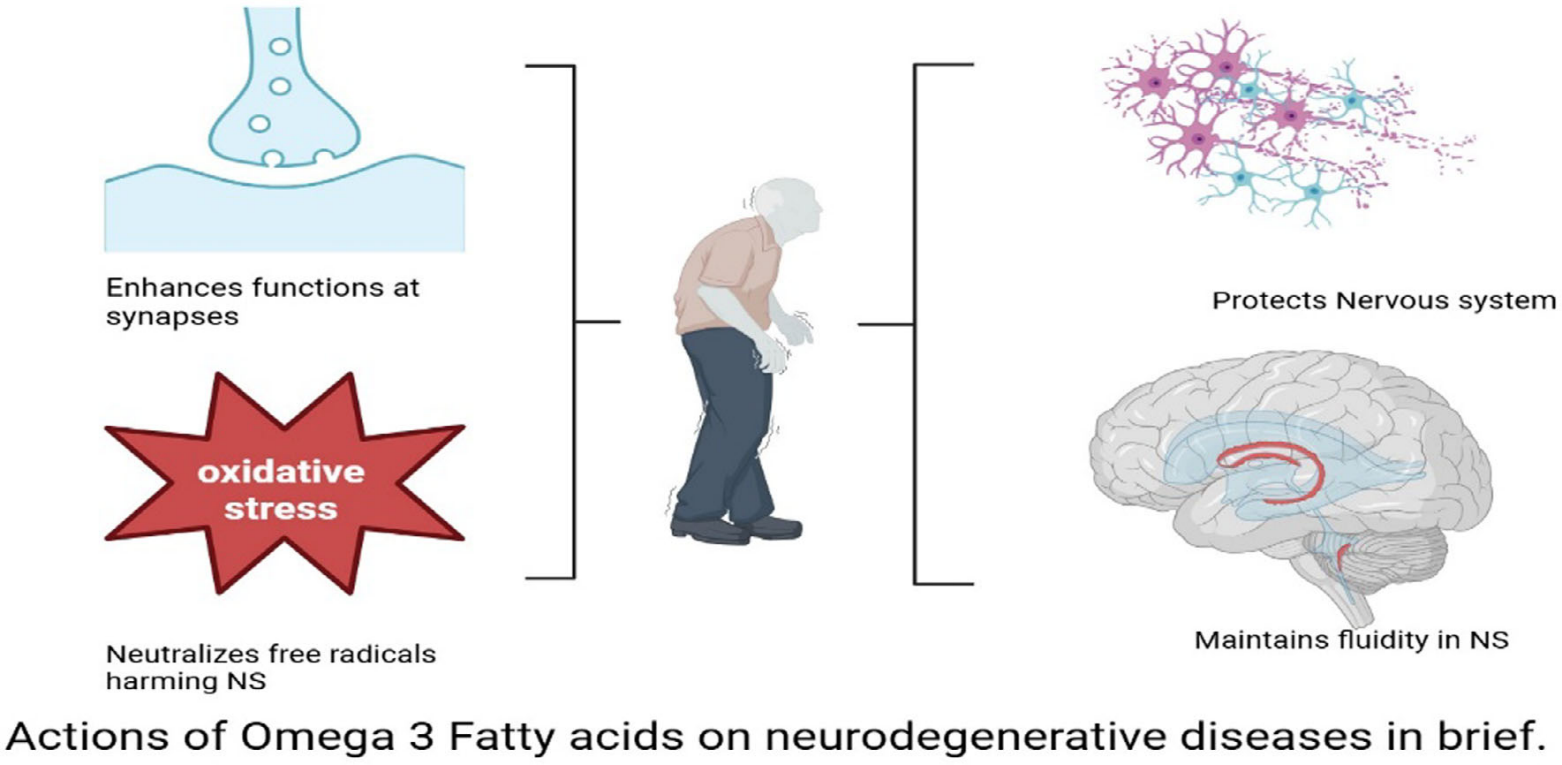
Omega‐3s are essential for cognitive health and could potentially provide protection against neurodegenerative disorders.

Alzheimer's disease (AD is a progressive neurological disorder. It mostly impacts cognitive and memory function. It is the most prevalent cause of dementia and is typified by a significant enough deterioration in thinking, memory and social skills to interfere with day‐to‐day functioning.^228^, ^229^ Many articles recommend the use of omega‐3s in AD. One research study conducted on fish and seafood supplements suggests that individuals who are on omega‐3s have a lower risk of developing AD.^230^ Another study reported that a person who was using omega‐3s more often had less cognitive decline compared to those who were not using it.^231^ Some studies suggest that people who use omega‐3s rich food have less age‐related dementia than those who are not using it, and this is because omega‐3s have anti‐inflammatory properties; hence, there is reduced brain inflammation.^232^ It also has a neuroprotective effect because of its amyloid deposition. Amyloid plaques in the brain, which slow down thinking and memory, are prevented. Also, because they are antioxidants, they can help fight harmful radicals that cause oxidative stress and damage to brain cells.^233^, ^234^, ^235^ Overall, omega‐3s might not have a significant impact on AD patients’ cognitive loss. Numerous studies have demonstrated that using omega‐3 supplements does not significantly improve cognitive performance in persons with mild to moderate AD.^232^, ^236^ Nevertheless, some research indicates that omega‐3 supplements may help reduce cognitive deterioration in relatively mild cases of AD.^237^ According to certain research, omega‐3s may help AD patients delay their cognitive and functional impairment, particularly when used in conjunction with other supplements such as alpha‐lipoic acid. Nevertheless, the evidence varies throughout studies, and more investigations are required to validate these results.^232^ Omega‐3s have been linked to reduced oxidative stress and inflammation, both of which play important roles in AD.^236^ The variation in study results emphasizes the need for more investigation to fully comprehend the circumstances in which omega‐3 supplementation may be advantageous, including dietary variables and illness stage. Common mechanisms of neurodegenerative disorders depicted in Fig. 5.

#### Mental health disorders

##### Bipolar disorder

Bipolar disorder comprises another behavioral disease considered to occur as a result of neuroinflammation. It is characterized by alternating episodes of mania/hypomania and depression. It is not easy to diagnose bipolar disorder because it coexists with other medical problems or psychiatric conditions.^238^ It is speculated that bipolar disorder occurs because of certain genetic conditions.^239^ Treatment of bipolar disorder is complex and requires extensive observation of the patient. Sometimes, major depression transitions into bipolar disorder; because of this, treatment becomes extremely challenging.^240^ Sometimes, patients may need maintenance treatment for long period or even over a lifetime to achieve control of symptoms.

Omega‐3s are essential for brain function. It has been found that they keep fluidity in the nervous system. They have anti‐inflammatory action, reduce inflammation and improve function at synapses, as well as exhibit potential function in mood stabilization, bipolar disorder and depression.^241^, ^242^ Omega‐3s have demonstrated some potential in lowering depressive symptoms in bipolar disorder, especially when used as an adjuvant treatment. Although the results vary and are not always significant, a number of studies and meta‐analyses show a moderate effect on bipolar depression. Many clinical guidelines support using of omega‐3s as supportive treatment in bipolar disorder.^243^, ^244^ Although their effectiveness in treating manic symptoms of bipolar disorder is not well established, omega‐3s may be somewhat helpful in treating depressed symptoms. To define precise clinical criteria and comprehend the underlying mechanisms, further study is required.

##### Schizophrenia

Schizophrenia is a severe mental condition that influences an individual's cognition, emotions and behavior. Individuals diagnosed with schizophrenia may exhibit a variety of symptoms such as hallucinations, delusions, disorganized thinking and speech.^245^ Negative symptoms include feeling a lack of motivation and not feeling happiness. Cognitive symptoms include impaired memory and impaired higher functions, such as an inability to make decisions, difficulty focusing, etc. Treatment of schizophrenia is time‐consuming and difficult, and some of its types, such as disorganized schizophrenia, do not respond to any treatment. Patients typically require antipsychotic drugs.^246^, ^247^

Research suggests that omega‐3s may help lower the intensity of symptoms in people with schizophrenia, especially those who are experiencing their first episode. The Positive and Negative Syndrome Scale (PANSS) showed a substantial improvement in symptom severity following a 26‐week omega‐3 supplementation intervention. Studies have produced conflicting findings, nevertheless, with some trials finding no discernible effect on acute schizophrenia patients' symptoms or animosity.^248^, ^249^ In animal models, omega‐3 supplementation during adolescence has been demonstrated to prevent behavioral deficits and brain abnormalities associated with schizophrenia, indicating a preventive effect against the development of such deficits.^250^ Omega‐3s have been linked to improvements in depression symptoms and overall psychopathology in humans.^248^

##### Dementia

**Figure 6:**
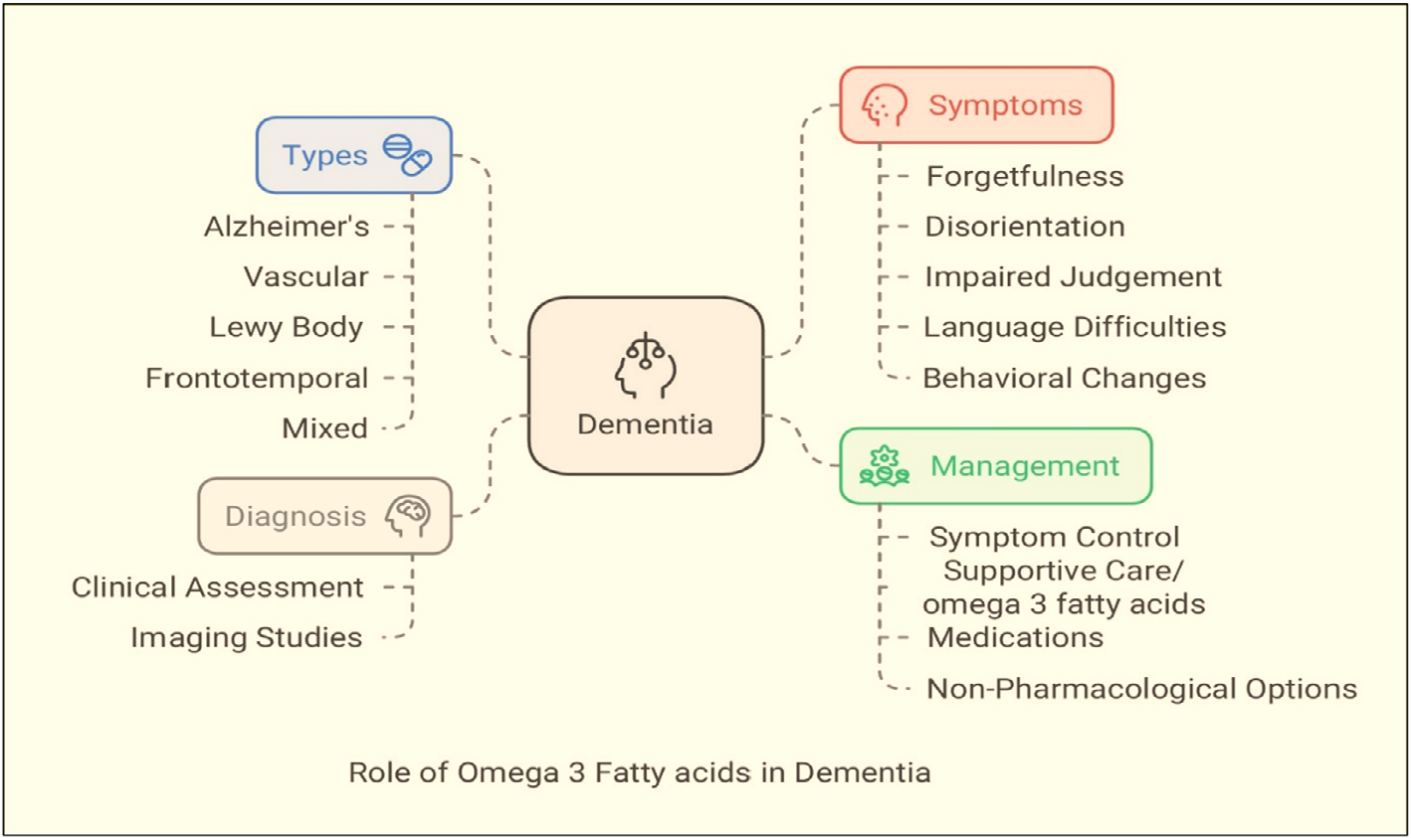
Various symptoms and types of dementia and role of omega‐3s fatty acids in the management of dementia.

Dementia is a general term for an impairment in cognitive function that interferes with daily activities. It is a group of symptoms that can be caused by a number of underlying illnesses, including Lewy body dementia, vascular dementia and AD. Memory loss, communication problems, poor reasoning and judgment, and behavioral or emotional changes are all possible symptoms of dementia.^251^, ^252^, ^253^ Similar to other degenerative diseases, omega‐3s especially EPA and DHA are considered very beneficial in dementia. This does not usually help in curing the disease, but its action is supportive, and it delays disease progression. Some studies suggest that those omega‐3s which are found abundantly in brain cells, increase the fluidity and improve the synaptic function. Some scholars report that omega‐3s reduce the inflammation in the brain cells, decrease the oxidative stress and reduce beta amyloid deposition, which is considered the most common cause of dementia.^254^, ^255^ There are some studies that suggest omega‐3 supplementation delays cognitive decline in mild cognitive impairment and the early stage of AD.^256^ A brief summary is depicted in Fig. 6.

### Pregnancy

Maternal diet significantly impacts the health of both mothers and their offspring. The maternal health and the nutritional condition of the child throughout the initial 1000 days of life, commencing at conception and extending through the second year, are crucial for ensuring good physical, mental and social growth, as well as development and lifetime health.^257^, ^258^ Therefore, for optimal growth and development throughout the first 1000 days, all necessary components including those from the diet must be present in adequate amounts during important phases of development.^258^ Commonly consumed nutrients through foods that are essential throughout this initial period of rapid growth are omega‐3s, particularly EPA and DHA, which have numerous health benefits.^258^ Supplementation of omega‐3s during pregnancy is linked to increased levels in the brain, central nervous system (CNS) and visual system of the developing fetus. Furthermore, they contribute to the duration of gestation and the prevention of perinatal depression.^259^ The placenta transports nutrients, such as DHA, from the mother to the fetus throughout gestation. The concentration of omega‐3s in the fetus is directly proportional to the intake by the mother, ensuring optimal maternal nutrition. Accumulation of the DHA in the fetus tissue is higher during the third trimester. Several studies have confirmed the beneficial effect of omega‐3 supplementation for the period of pregnancy in terms of proper development of the brain and retina and association with a longer gestation period.^260^, ^261^, ^262^

Another study by Masot *et al*. (2023) reported that lower level of omega‐3s in pregnant women was significantly associated with depression and anxiety, Consequently, DHA may be valuable in the prevention of the onset of anxiety and depression during pregnancy.^139^ Moreover, an adequate omega‐3s supply throughout pregnancy and breastfeeding is linked to fetal brain and retina development, a lower incidence of preterm birth, and a diminished risk of gestational diabetes, pre‐eclampsia and post‐partum depression (PPD).^263^, ^264^ PPD is a common mental health issue affecting mothers, and low omega‐3s levels particularly DHA and EPA have been interrelated to enhancing the risk of PPD. Omega‐3s exhibit a role in the regulation of mental health, and their deficiency in pregnancy and lactation may contribute to depressive symptoms. Several studies recommend that supplementation with EPA‐rich or DHA‐rich oils during pregnancy can decrease the threat of PPD, mainly in women with low baseline omega‐3 levels^247^ ^.^ ^247^, ^265^, ^266^ Overall, continuing sufficient omega‐3s consumption during pregnancy may help lower the risk of PPD, but supplementation exhibits the most benefit for women with low omega‐3s levels or those having a history of depression.^264^, ^266^

DHA is required for the maintenance of homeostasis of the retina and for photoreceptors to function properly. The proper development and function of the retina depend on an adequate supply of long‐chain polyunsaturated fatty acids. DHA is an important retinal structural lipid, constituting up to 50% of the total lipid content in photoreceptor rod outer segments.^267^ The alteration in ideal DHA levels can influence membrane fluidity, hence impacting the activity and regeneration of rhodopsin, which eventually affects phototransduction.^268^, ^269^ Furthermore, adequate DHA intake during pregnancy and the early years of life is essential for the proper visual and cognitive development of the child.^270^

Omega‐3s have beneficial effects on a variety of organs and biological processes, including growth regulation and platelet activation.^271^ A constant supply of omega‐3s (DHA and EPA) is required for the functioning and formation of the structure of the brain, it accumulates predominantly in the fetal brain throughout the third trimester of gestation and persists at elevated rates of accumulation until the second year of life.^261^, ^272^ DHA serves as a structural element of membranes within the CNS. Throughout pregnancy, half of the DHA in the brain is synthesized because the developing infant brain necessitates five times the lipid intake daily in contrast to an adult brain. The production of DHA by the fetus and placenta is not sufficient to meet the demands of rapidly growing neural structures, and hence maternal DHA levels are critical for the development of the fetal brain.^272^ Omega‐3s are known for their anti‐inflammatory effects through mechanisms such as the formation of specialized pro‐resolving mediators. DHA and EPA release prostaglandins in the endometrial cells. Furthermore, molecules such as cyclooxygenase‐2, IL‐1β, NF‐κB and intracellular signaling pathways are also affected by omega‐3s in endometrial cells.^273^ Additionally, omega‐3s are also associated with the prevention of preeclampsia, which is a multisystem disease, unique to human pregnancy, characterized by elevated blood pressure and proteinuria.^274^ This disorder is mostly prevalent during pregnancy and can lead to substantial fetal, neonatal, and maternal morbidity and mortality.^275^ Fatty acids cannot be produced independently; hence they are crucial for the fetus's development throughout pregnancy. During the prenatal period, there is a decrease in the maternal tissue store for fatty acids; hence, omega‐3 fatty acid supplementation is often recommended during pregnancy. Li *et al*.^272^ and Bakouei *et al*. (2020) have reported that the consumption of omega‐3s protects pregnant women against preeclampsia. Studies have reported that supplementation with omega‐3s prevented the incidence of preeclampsia in women with low‐risk pregnancies.^272^, ^276^

**Figure 7:**
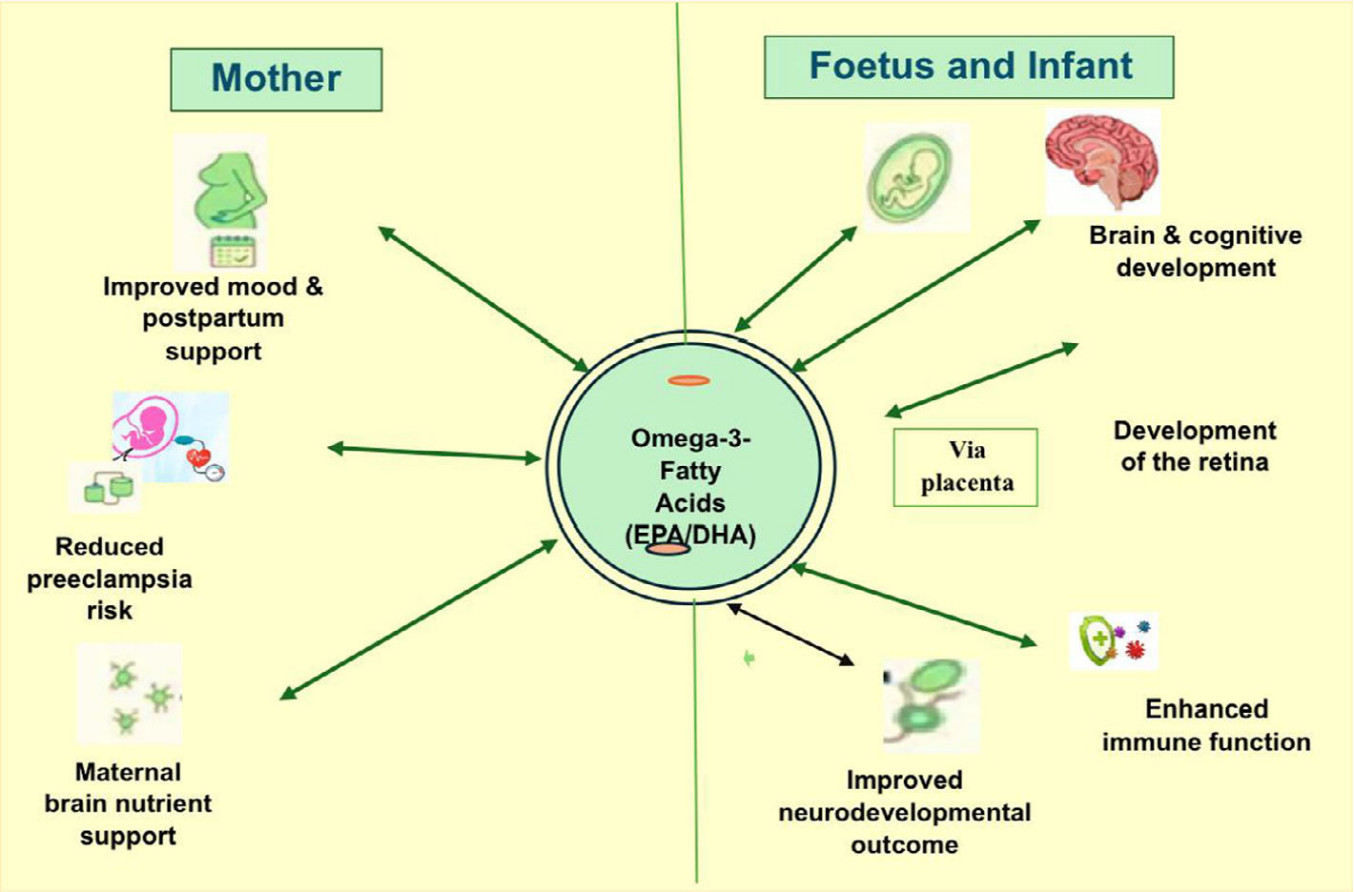
Omega‐3s potential role in maternal and fetal development.

Gestational diabetes mellitus is linked to heightened health risks for both the mother and fetus during pregnancy. Timely identification of gestational diabetes will enhance the health of both mother and fetus.^277^ It is a typical consequence of pregnancy linked to poor glucose metabolism and insulin resistance, resulting in elevated blood sugar levels.^278^ The risk of developing type 2 diabetes mellitus and coronary heart disease is significantly heightened in individuals with gestating diabetes, which may also cause a rise in inflammatory cytokines and maternal insulin concentrations, resulting in insulin resistance.^279^ Furthermore, because of the limited synthesis of fetal omega‐3s and their transfer to the fetus through the placenta, the quantities and composition of these fatty acids fundamentally depend on the metabolic condition of the mother.^280^ Pregnancy causes changes in the mother's lipid and fatty acid metabolism to support the growth and development of the fetus. Accordingly, taking supplements of omega‐3s can have a significant positive impact on glycaemic management and the inflammatory response among individuals with gestational diabetes.^281^ Hence, supplementation with omega‐3s in pregnant women could have a positive influence on the health of both the mother and the fetus (Fig. 7).

### Lower the risk of preterm birth

DHA has been broadly studied for its protagonist role in decreasing the preterm birth (PTB) risk, chiefly early preterm birth (EPTB). Various randomized controlled trials and meta‐analysis have reported that supplementation with DHA throughout the pregnancy, particularly at higher doses is linked with a substantial reduction in the rates of EPTB and PTB. A remarkable advantage was noted in women who have low level at the start of pregnancy.^282^, ^283^, ^284^ The anti‐inflammatory properties of DHA are likely to be intricate in the mechanism. These characteristics help to regulate the immunological responses and diminish inflammation‐driven pathways, which can cause early labour.^285^, ^286^ Supplementation timing as well as the dosage are very important. Some evidence suggests that the most effective way to take DHA supplements is to start taking them before week 20 of pregnancy and to continue taking them until at least weeks 34–37 of pregnancy.^282^ Cetin *et al*.^287^ reported in their study that women of reproductive age should acquire a minimum of 250 mg day^–1^ of DHA and EPA from dietary sources or supplements, whereas, in pregnancy, an extra intake of ≥ 100 to 200 mg day^–1^ of DHA is recommended. Pregnant women with decreased DHA consumption and/or low DHA serum levels exhibit a heightened risk of preterm and early preterm birth. Supplementation of DHA and EPA should commence in the second trimester of pregnancy and persist until almost week 37 of pregnancy or until delivery.^287^ Women having low DHA levels get the most benefits because studies show a reduction of EPTB rates within this demographic when administered high‐dose DHA.^284^, ^288^ On the other hand, women with adequate DHA levels may not gain further advantages from supplementing, and over‐supplementation in adequately supplied women may potentially raise risk.^282^, ^288^ Pregnant women with insufficient docosahexaenoic acid consumption and/or low blood levels of docosahexaenoic acid face an increased risk of preterm and early preterm birth. Consequently, patients should be administered a dosage of about 600–1000 mg day^–1^ of DHA and EPA, or just DHA, because this dosage has shown a significant reduction in preterm and early preterm births in randomized controlled trials.^283^, ^287^ DHA supplementation throughout pregnancy is beneficial in reducing the risk of preterm birth for women who have a poor DHA status. This beneficial effect is especially true when the supplement is taken in higher doses and when it is started at an earlier stage. Increasing the benefits and improving the pregnancy outcomes can be achieved through screening for DHA levels and targeted supplementation.

### Fetal brain and retinal development

DHA is an omega‐3s fatty acid that acts as a major structural component of the brain and retina. Accumulation of DHA is more prominent in the last trimester of pregnancy and it remains at a persistent level during early development. It promotes synaptogenesis, neurogenesis and maturation of visual system. DHA abundantly present in the gray matter of the brain and retinal photoreceptors, where it enables membrane fluidity, neurotransmission and synaptic plasticity. DHA promotes gene expression of gene linked with neurogenesis, and its deficiency may hinder visual function and neural development.^289^, ^290^, ^291^, ^292^ DHA maintains the integrity of the blood–retinal barrier and blood–brain barrier through protecting against oxidative stress.^268^, ^293^ The fetus depends on maternal DHA, which is transported through the placenta. Accordingly, DHA levels in the mother directly affect fetal brain and retinal development.^294^ Long‐term studies on prenatal DHA supplementation highlight that it decreases the preterm delivery risk and improves visual attention during infancy.^295^ Moreover, maternal diets rich in omega‐3s, mainly DHA, have confirmed the ability to enhance the fetal brain development and cognitive function and raise the membrane fluidity, which are essential for optimal brain function.^296^, ^297^ In the retina, DHA uptake is enabled by particular transporters such as Mfsd2a, which is essential for the development of photoreceptors.^298^ DHA also regulates the expression of genes in the developing retina, particularly genes that involved in neurogenesis and synaptic connectivity.^291^ Overall, the influence of DHA on fetal brain and retinal development is facilitated by complex molecular mechanisms, and its role in early developmental benefits is well‐supported.

### Lactation

The nutritional status of a mother is a significant factor that influences the birth of a child and the child's subsequent health outcomes during the later period of life. The optimal source of nutrition that an infant requires for growth and development is breast milk. Various studies have reported that infants had better neurodevelopment when they were breastfed than formula‐fed infants, and this advantage is a result of the presence of omega‐3s, especially DHA in the breast milk,^299^ This is also supplemented in the majority of pregnant and lactating women by the normal vitamin regimen.^300^ Hence, omega‐3s is important for the development of the infant and also for the health of the mother. The concentration of omega‐3s in the mother's milk, which is an optimal source of nutrition for infants for the first 6 months, is determined by the maternal blood levels, which are influenced by the intake. DHA provides significant health benefits for lactating mothers. Studies have shown that babies who were fed breast milk with a higher DHA content had improved neurodevelopmental and visual outcomes.^301^ Maternal nutrition during lactation has a significant impact on the child's growth and long‐term health. Omega‐3s are particularly important for myelination and the development of vision during the perinatal period.^257^

Fatty acids are mostly obtained from the mother because infants cannot synthesize them during the first few days of life. During pregnancy, the fetus receives omega‐3s through the placenta and umbilical cord blood, and after the birth through breast milk. Some 70% of omega‐3s in breast milk originate from the stored fatty acids in the adipose tissue of the mother's body during pregnancy and the remaining 30% is through supplements and diet. For infants, the first 2 years of life are crucial for neurodevelopment. DHA plays numerous vital functions in the brain, such as controlling gene expression, neurotransmission and cell signaling.^290^, ^302^, ^303^, ^304^ Additionally, supplements raise the amount of omega‐3s in breast milk, especially during the early stages of nursing, which is positively correlated with the infant's DHA levels for up to 1 year.^290^, ^305^ DHA is important for infants’ visual and cognitive development because DHA is an essential component of photoreceptors and membranes of the brain cells.^306^

For the development of a healthy newborn mammalian, DHA and EPA are necessary. Omega‐3 supplementation for a period of 9 months, starting from the last trimester to the sixth month of lactation, considerably increases the levels of DHA and EPA in mature milk and colostrum.^307^ The tissue levels of fatty acids during lactation are closely related to mother's storage capacity and the metabolic use of fatty acids (synthesis, oxidation and transport). Hence, the metabolism of fatty acids and the diet of a lactating mother is an important factor that affects the concentration of DHA in the breast milk. The fatty acid composition of breast milk changes continuously as dietary fatty acids is rapidly transported from chylomicrons into the breast milk, with a peak between 6 and 12 hours after the intake of DHA.^308^ The mental well‐being of the lactating mother requires omega‐3s, which is associated with the neuroprotective and anti‐apoptotic action, and has an antidepressant effect.^309^, ^310^, ^311^ Myelination, neurogenesis, synaptogenesis, neurotransmitter metabolism, cell differentiation, neuronal migration and inflammatory responses are all processes that are affected because of the deficiency of omega‐3s. DHA and EPA also decrease the levels of cytokines and depressive symptoms, as well as the risk of food allergies and depression. Adequate doses of omega‐3s are important to prevent deficiencies and for prophylactic purposes in pregnancy and lactation.^312^

### Antidiabetics

The potential role of omega‐3s, particularly their impact on diabetes prevention and control, has been thoroughly investigated.^313^, ^314^ ALA, EPA and DHA have antidiabetic benefits via a number of pathways.^315^ Insulin sensitivity is one of the main ways that omega‐3s may help people with diabetes. Increased insulin sensitivity lowers blood sugar levels by improving the body's cells’ ability to use glucose.^316^ By activating peroxisome proliferator‐activated receptor gamma (PPAR‐γ), omega‐3s promote the expression of plasma adiponectin and leptin, therefore improving insulin sensitivity by means of glucose transporter type 4 (GLUT‐4) receptor upregulation. Omega‐3s lower the fat content in the muscle, preserve normal function of phosphoinositide 3‐kinase and increase GLUT‐4 receptor synthesis and transcription in the muscle, thereby enhancing glucose absorption. Omega‐3s can directly act as anti‐inflammatory agents and reduce insulin resistance by improving inflammation in adipose tissue.^316^, ^317^, ^318^, ^319^

It has been demonstrated that omega‐3s increase the activities of antioxidant enzymes, which can lower oxidative stress, a major contributing factor to the development of diabetes.^31^ Omega‐3 supplementation in diabetes has been linked to lower levels of lipid peroxidation and higher activity of glutathione peroxidase, a vital antioxidant enzyme.^320^ The body's antioxidant capacity may be strengthened by omega‐3s, which may lower the risk of vascular problems in diabetics.^321^ Although preclinical and some clinical research has indicated that omega‐3s have potential, it is still difficult to translate these advantages into reliable clinical results in people. Although some studies demonstrate modest benefit, others report positive effects on glucose metabolism and insulin sensitivity.^322^, ^323^

The gut microbiota's composition and action are influenced by omega‐3s, specifically EPA and DHA.^26^ They encourage the growth of healthy bacteria, such as *Lactobacillus* and *Bifidobacterium*. They decrease the number of harmful and pro‐inflammatory microorganisms. They increase the synthesis of butyrate and other short‐chain fatty acids (SCFAs). G‐protein‐coupled receptors (GPCRs) are activated and glucose homeostasis is improved by increased SCFA synthesis.^324^, ^325^ Type 2 diabetes, inflammation and insulin resistance are linked to gut microbiota imbalance.^325^, ^326^ This is countered by omega‐3s, which decrease intestinal permeability and restore microbial diversity. Lipopolysaccharides (LPS) and other endotoxins may be prevented from entering the bloodstream by omega‐3s.^21^, ^324^, ^327^, ^328^ Because of their anti‐inflammatory and immune‐modulating qualities, omega‐3s may help preserve the integrity of the intestinal barrier. Endotoxins are a major cause of inflammation and insulin resistance, and an optimal gut barrier helps keep them out of the bloodstream.^324^, ^329^

**Figure 8:**
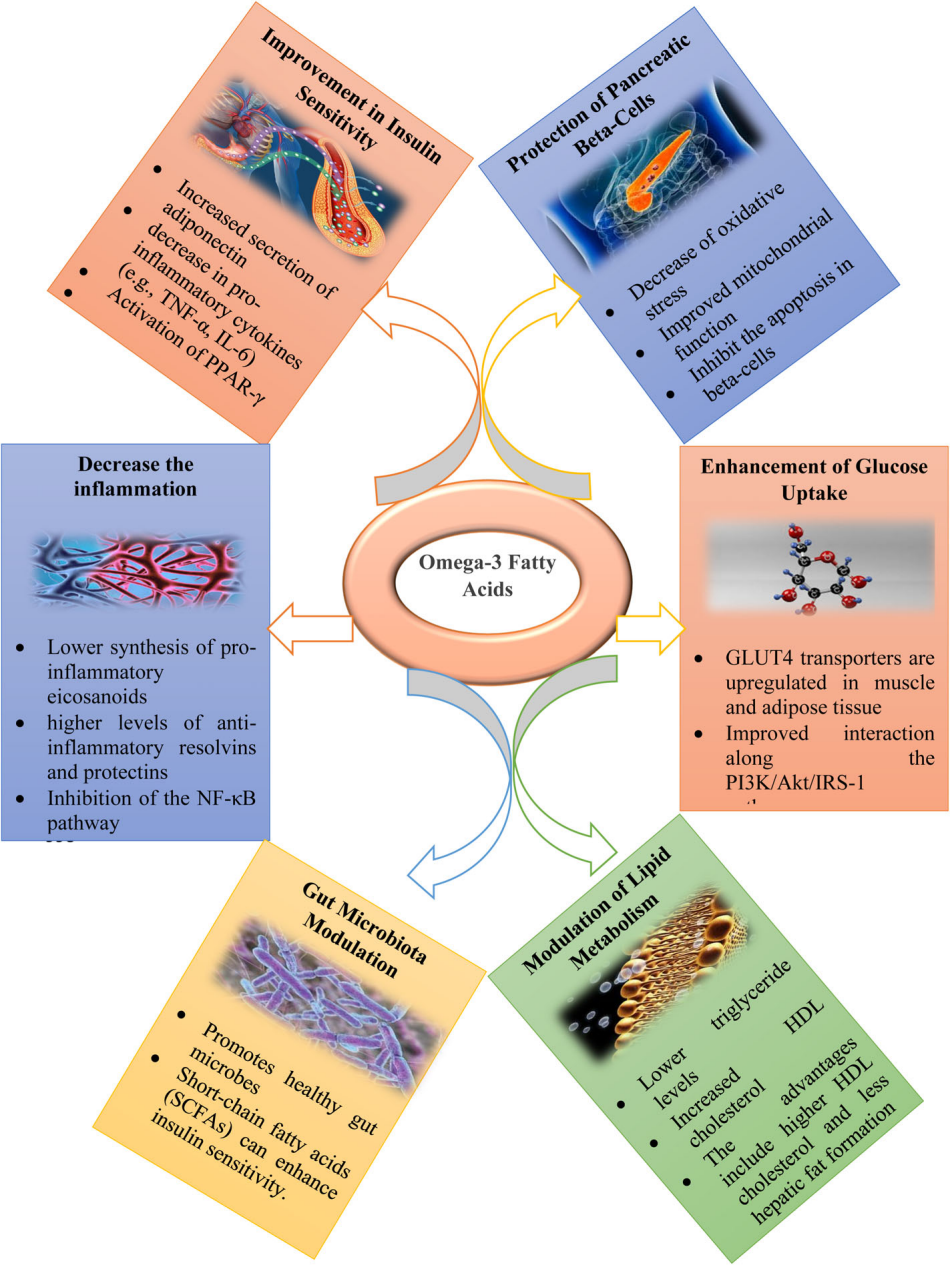
Various mechanisms exhibited by omega‐3s against diabetes.

Additionally, the results of randomized controlled trials conducted with people who have been diagnosed with type 2 diabetes regarding glycemic control are ambiguous. Upon analyzing the data, it was discovered that there were no noteworthy variations in the HbA1c levels in four of the studies,^317^, ^330^ fasting blood glucose (FBG) levels in five of the studies or homeostatic model assessment for insulin resistance (HOMA‐IR) levels in four of the studies.^330^, ^331^, ^332^, ^333^ Five studies reported significant drops in HOMA‐IR levels,^334^, ^335^, ^336^, ^337^ five studies reported significant drops in FBG levels^334^, ^338^ and four studies reported significant drops in HbA1c levels.^334^, ^338^, ^339^ According to the study, the group that consumed omega‐3s with a low‐carb, high‐protein diet saw a larger drop in HbA1c levels.^338^ This reinforces the need for dietary therapy in managing diabetes. This disparity highlights the necessity of more studies aiming to completely comprehend the causes and maximize the application of omega‐3s in the treatment of diabetes (Fig. 8).

### Anti‐inflammatory

Omega‐3s, EPA and DHA, are prominent anti‐inflammatory agents. These long‐chain polyunsaturated fatty acids have an important role in moderating inflammatory processes.^92^ Omega‐3s, particularly EPA and DHA, exhibit their anti‐inflammatory activities through targeting definite cell signaling pathways and cytokines essential to the inflammatory process. NF‐κB one of the most significant pathways. NF‐κB is a transcription factor that, when triggered, migrates into the cell nucleus and promotes gene expression encoding pro‐inflammatory cytokines that include TNF‐α and IL‐6. Through inhibiting phosphorylation and degradation, omega‐3s inhibit the activation of NF‐κB by preventing the phosphorylation and degradation of its inhibitor (IκB). Thus decreasing the inflammatory transcription genes and cytokines production such as TNF‐α as well as IL‐6.^43^, ^340^, ^341^, ^342^ Moreover, omega‐3s can trigger PPAR‐γ, which further decreases action of NF‐κB and expression of inflammatory gene.^341^, ^343^ Additionally, the key path is via the enzyme COX‐2. It generates pro‐inflammatory prostaglandins from arachidonic acid (omega‐6 fatty acid). Omega‐3s compete with arachidonic acid for COX‐2 and produce a lesser amount of prostaglandins and particular pro‐resolving mediators such as resolvins and protectins, which resolve inflammation. Through these mechanisms, omega‐3s decrease pro‐inflammatory cytokine levels such as TNF‐α IL‐6 and IL‐1β.^21^, ^43^, ^341^, ^342^, ^344^ EPA and DHA are precursors to specific pro‐resolving mediators, including resolvins, protectins and maresins. These mediators play an important function in treatment of inflammation by inhibiting neutrophil recruitment and improving inflammatory cell clearance.^21^, ^327^, ^345^, ^346^ Moreover, the phospholipids in cell membranes are altered by omega‐3s, which cause the synthesis of eicosanoids to change from pro‐inflammatory to less inflammatory or anti‐inflammatory forms. This change is linked to a reduction in the synthesis of eicosanoids and pro‐inflammatory cytokines that are produced from arachidonic acid.^21^, ^327^, ^347^

**Figure 9:**
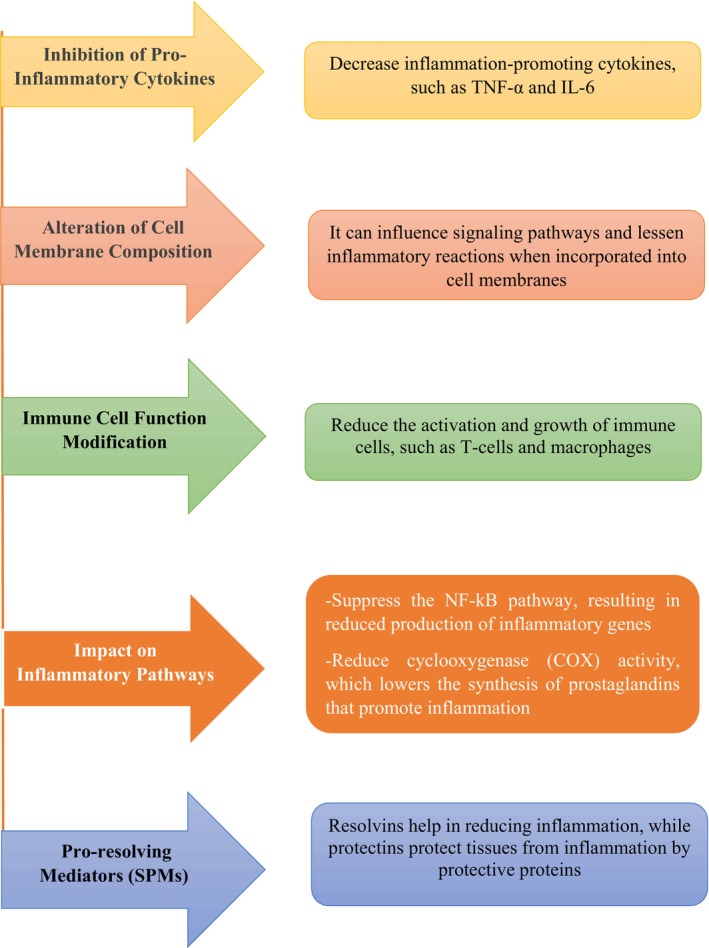
Omega‐3s are essential in mitigating inflammation *via* several mechanisms: suppressing pro‐inflammatory cytokines, enhancing anti‐inflammatory cytokines, modifying cell membrane composition and decreasing oxidative stress.

Asthma, psoriasis, rheumatoid arthritis and inflammatory bowel problems are among the chronic inflammatory diseases that omega‐3s have demonstrated therapeutic promise in treating. They are helpful in lowering symptoms and slowing the progression of disease because of their capacity to alter inflammatory pathways.^348^, ^349^, ^350^ This makes them especially advantageous for joint health because research has indicated that omega‐3s might lower the pain and stiffness associated with rheumatoid arthritis.^351^, ^352^ By lowering the risk factors linked to atherosclerosis and other cardiovascular disorders, the anti‐inflammatory properties of omega‐3s promote cardiovascular health. They reduce blood pressure, enhance endothelial function and lower TGs. Pre‐clinical models of neurodegenerative and mental illnesses have demonstrated the anti‐inflammatory benefits of omega‐3s and their metabolites. By altering inflammatory cytokines and signaling pathways, they enhance brain function and decrease depressive‐like behaviors.^350^, ^353^ In conclusion, omega‐3s have substantial anti‐inflammatory properties that are relevant to a variety of chronic illnesses because of their varied modes of action (Fig. 9). Their capacity to regulate inflammation underscores their potential as therapeutic agents in clinical and prophylactic contexts. It is still difficult to determine how much and how to administer omega‐3s to obtain the greatest anti‐inflammatory effects. To establish uniform guidelines for their usage in different inflammatory diseases, more clinical trials are required.^350^

### Hepatoprotective

**Figure 10:**
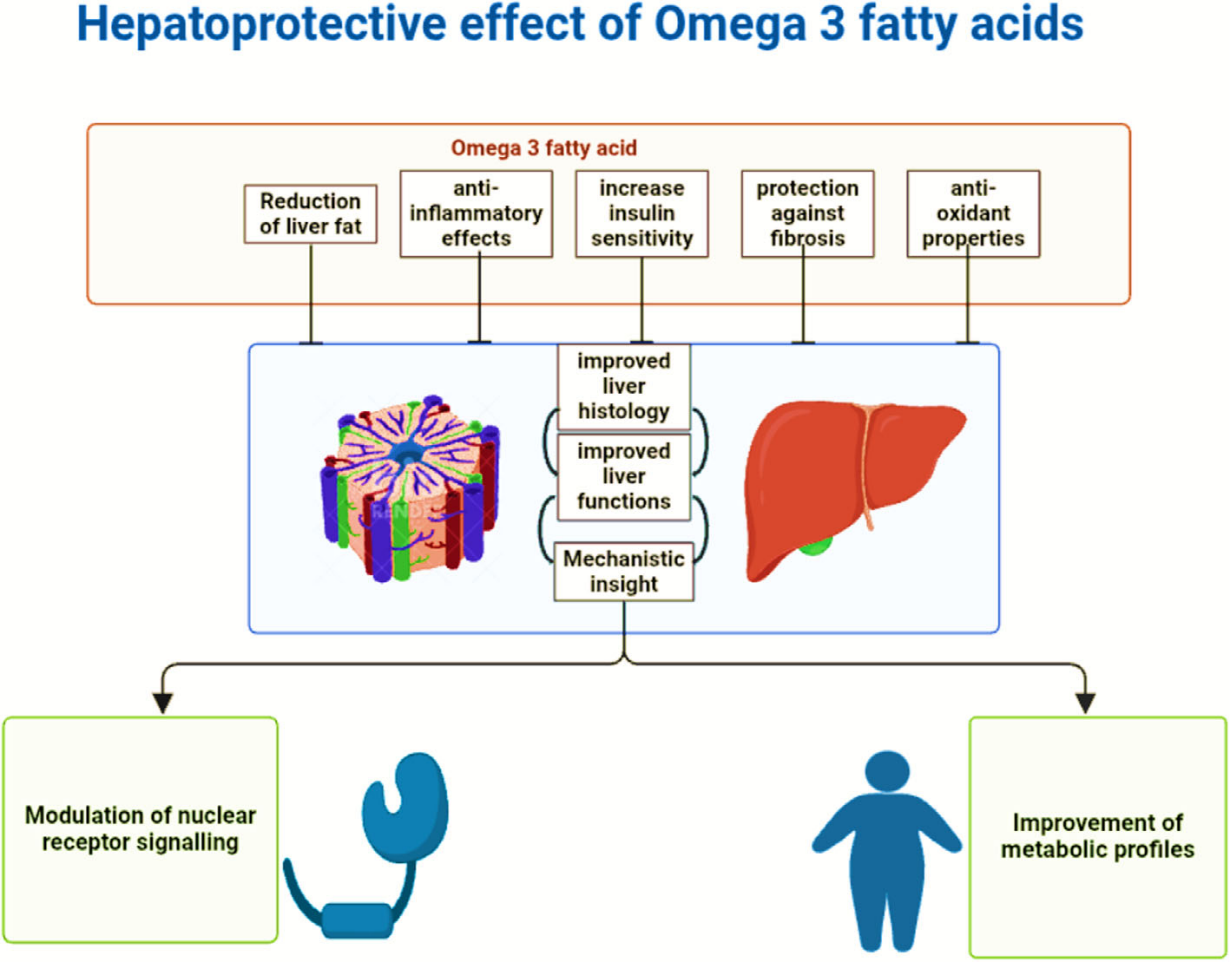
Hepatoprotective effects of omega‐3s through multifaceted mechanisms such as increased insulin sensitivity, decreased hepatic fats, inflammatory effects and potentiating antioxidant properties.

Omega‐3s have been found to be effective in liver disorders in many ways. They modulate lipid metabolism, reduce inflammation and improve liver function. These compounds decrease liver fat content in individuals with non‐alcoholic fatty liver disease.^85^, ^354^, ^355^ Omega‐3s also have anti‐inflammatory properties, in that they inhibit inflammatory cytokines and modulate the immune response, which also plays a critical role for liver inflammation and hepatitis.^193^ Omega‐3s improve insulin sensitivity, therefore reducing hepatic glucose production and lowering liver stress. Omega‐3s help to prevent the transition of liver fibrosis into metabolic disorder.^356^, ^357^ Omega‐3s activate hepatic stellate cells, which are responsible for apoptosis of activated fibroblasts. These fibroblasts eventually produce liver fibrosis. Studies suggest that omega‐3s may help in prevention of liver fibrosis because they stimulate apoptosis of liver fibroblasts.^357^, ^358^ Some studies show that supplementation of treatment with omega‐3s in patients with non‐alcoholic steatohepatitis improves liver histology, and reduces inflammation and fibrosis.^359^ Hepatoprotective effects are depicted in Fig. 10.

Furthermore, omega‐3s strengthen the antioxidant protection mechanisms in the liver, thereby alleviating oxidative stress, which is frequently enhanced in hepatic disorders.^360^ They enhance insulin sensitivity, which is advantageous in averting hepatic disorders linked to metabolic syndrome.^88^, ^361^ Studies indicate that omega‐3 supplementation can significantly reduce liver fat and enhance liver function tests in people with non‐alcoholic fatty liver disease.^362^, ^363^ Moreover, omega‐3s are proposed to impede the growth of liver fibrosis and may confer positive impacts to chronic hepatitis patients by enhancing hepatic inflammation and function.^362^, ^364^ Researchers also suggest the antioxidant properties of omega‐3s protect liver cells from oxidative stress and lipid per‐oxidation, which can lead to liver injury. Some studies suggest that even omega‐3s are helpful in obstructive jaundice, which is one of the main reasons for hepatic injury.^365^, ^366^ Integrating omega‐3s rich meals, including fatty fish, flaxseeds and walnuts, or applying supplements can effectively improve liver function and mitigate liver‐related disorders.^367^

## FUTURE OF OMEGA‐3S AS A FUNCTIONAL FOOD

The future prospective of omega‐3s as functional foods seems optimistic, fueled by heightened consumer knowledge of health benefits, innovations in production methods and continuous study into their medicinal uses.^57^ The global omega‐3s market is expected to widen considerably, with forecasts signifying a growth from over USD 2.10 billion in 2020 to nearly USD 3.61 billion by 2028. This growth is determined by heightened consumer interest in health and wellness, subsequent to the greater consumption of omega‐3‐enriched foods and supplements.^368^ The search for functional foods that offer health benefits beyond basic nutrition is propelling novelty in this industry. Developments in omega‐3sources are confronting sustainability issues and extending market prospects. Algal oil, a sustainable and plant‐derived source of DHA and EPA, is becoming gradually favored, particularly within vegan and vegetarian markets. Biotechnology advancements are facilitating the sustainable and economical manufacturing of omega‐3s. Technologies such as microbial fermentation employing genetically modified yeast and algae are being advanced to generate high‐grade omega‐3s independently of conventional fish sources, which encounter sustainability issues. This transition not only alleviates environmental concerns, but also improves the accessibility of omega‐3s for functional food applications.^49^, ^368^, ^369^, ^370^

Omega‐3s, namely EPA and DHA, are increasingly acknowledged for their extensive health advantages, encompassing cardiovascular health, cognitive function and anti‐inflammatory effects. Recent research and industry trends indicate a favorable outlook for omega‐3s, carried by scientific progress, sustainability efforts and increasing consumer awareness. Bhatt *et al*.^371^ reported that high‐dose EPA markedly diminishes cardiovascular events in high‐risk individuals, resulting in the formulation of prescription omega‐3 medications such as Vascepa (icosapent ethyl). With the growing public knowledge of nutritious meals, the market for omega‐3 supplements is expected to expand. Present sources comprise oily fish, algae and omega‐3s fortified functional foods. The projected development of novel dietary supplements employing omega‐3s in innovative manners is expected to improve their bioavailability and therapeutic efficacy.^372^ Regulatory organizations are progressively acknowledging the health benefits of omega‐3s, resulting in authorization for their use in numerous food products and supplements. This regulatory assistance is expected to promote additional innovation in the development of functional foods containing omega‐3s, hence enhancing their accessibility to consumers.^76^

**Figure 11:**
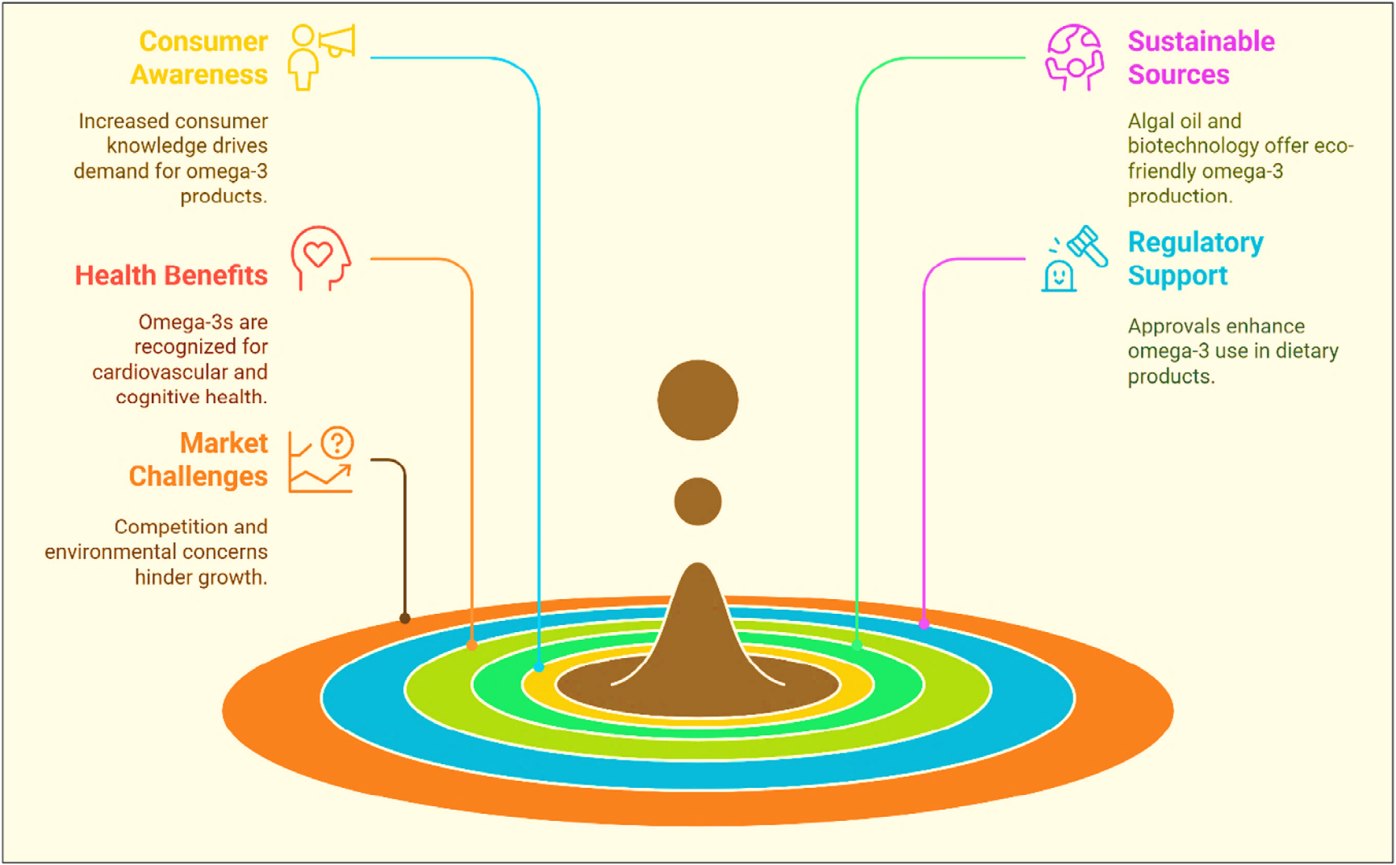
An illustration of the promising future for omega‐3s as functional foods, emphasizing market growth forecasts, innovations in sustainable manufacturing techniques and heightened consumer knowledge of health advantages. It underscores the transition to plant‐based sources, such as algal oil, and the contribution of biotechnology in augmenting the availability and effectiveness of omega‐3s for enhanced health results.

Alongside these developments, obstacles persist, including customer ambiguity on dosage and quality, competition from inexpensive supplements, and environmental issues associated with fish oil production. Nonetheless, other prospects exist, including the creation of high‐potency formulations, entry into emerging markets, and the incorporation of omega‐3s into functional foods and beverages. The future of omega‐3s is promising, propelled by sustainability, innovation and personalized nutrition, which are enhancing its applications in global health and well‐being (Fig. 11).

## CONCLUSIONS

In summary, omega‐3s are confirmed to be a functional food that offers a variety of health advantages. A potential role is revealed in heart disease, cancer, managing diabetes, pregnancy, lactation, CNS function and hepatic disorders, etc. Omega‐3s are essential for metabolism because of their functions in regulating cellular function, promoting heart health and reducing inflammation. The current review has emphasized the significance of omega‐3 supplements for the health of both mothers and infants. They have been associated with improved perinatal outcomes and extended gestational durations during pregnancy. During neonatal growth, omega‐3s are crucial for the CNS because they facilitate neuronal development and function. This process may have repercussions for cognitive health in maturity. However, the efficacy of omega‐3 supplementation can vary depending on the dosage, duration and individual health conditions. It is highly recommended to incorporate omega‐3‐rich foods into the diet. Novel formulations and applications for integrating omega‐3s into regular meals are essential for their future viability as functional foods. Novel omega‐3 formulations have been developed to improve bioavailability, stability and consumer appeal. Some examples comprise re‐esterified TGs that increase absorption efficiency, phospholipid‐based formulations that enhance cellular uptake and self‐emulsifying delivery systems that improve solubility in watery conditions. These formulations are mainly planned to address concerns such as oxidative instability, undesirable taste and odor, and limited absorption of traditional omega‐3 ethyl esters or TGs. Overall, omega‐3s continue to be a valuable component of a well‐balanced diet and function as a functional nutrient because they offer protection against a variety of health conditions.

## FUNDING

The Deanship of Graduate Studies and Scientific Research, Jazan University, Saudi Arabia, has financially supported through project number: RG24‐S090 to carry out this study.

## CONFLICTS OF INTEREST

The authors declare that they have no conflicts of interest.

## AUTHOR CONTRIBUTIONS

MFA was responsible for conceptualization. MFA, AAA, AK, AA, AR, FB, MZ, AOB, AFA, BM, MFB, MHA and MIA were responsible for investigations. MFA was responsible writing the original draft. MFA, AAA, AK, AA, AR, FB, MZ, AOB, AFA, BM, MFB, MHA and MIA were responsible for reviewing and editing. MFA, AR and MIA were responsible for visualization. AR and MIA were responsible for supervision. MFA and AR were responsible for funding acquisition. All authors have read and approved the final version of the manuscript submitted for publication.

## Data Availability

Data sharing not applicable to this article as no datasets were generated or analysed during the current study.
